# Supplementary material for: Leveraging ecosystems responses to enhanced rock weathering in mitigation scenarios
Source: Nat Commun. 2025 Mar 28;16:3021. doi: 10.1038/s41467-025-58284-6 (PMC11950449; doi:10.1038/s41467-025-58284-6)
Supplement: Supplementary file 1 — Supplementary Information [file 41467_2025_58284_MOESM1_ESM.pdf]

# Leveraging ecosystems responses to enhanced rock weathering in mitigation scenarios

Yann Gaucher[1,2,*], Katsumasa Tanaka[1,3], Daniel J.A. Johansson[4], Daniel Goll[1] and Philippe Ciais[1]

# Supplementary Information

**1. Enhanced weathering module** **3**
1.1 Carbon dioxide removal 3
1.1.1 Weathering rates 3
1.1.1.1 Weathering rate = 1% per year 6
1.1.1.2 Weathering rate = 25% per year 8
1.1.2 Calibration of the model 10
1.1.3 Long-term dynamics of the model 13
1.1.4 Spatial distribution of basalt application 15
1.2 Basalt supply 15
1.2.1 Transport distances 16
1.2.2 Cost of aircraft application 18
1.3 Break-even CO2 price of basalt application 20
1.3.1 Costs 20
Non-energy costs 20
Energy costs 21
1.3.2 Benefits 21
1.3.3 Hotelling rule and beyond 22
**2. Sensitivity Analysis** **23**
2.1 Parameters distributions 23
2.2 Latin hypercube sampling 27
2.3 Morris sampling 27
2.4 Discount rate 29
**3. GET-ACC2 model** **31**
3.1 Overview of the model 31
3.1.1 Optimisation procedure 32
3.1.2 Model Validation 33
3.2 Major updates on GET 34
3.2.1 Fossil fuel supply curves 34

[1] Laboratoire des Sciences du Climat et de l'Environnement (LSCE), IPSL, CEA/CNRS/UVSQ, Université Paris-Saclay, Gif-sur-Yvette, France
[2] CIRED, Ecole des Ponts, Nogent-sur-Marne, France
[3] Earth System Division, National Institute for Environmental Studies (NIES), Tsukuba, Japan
[4] Department of Energy and Environment, Chalmers University of Technology, Gothenburg, Sweden
* yann.gaucher@enpc.fr

3.2.2 Biomass supply curve 34
3.2.3 Methane and Nitrous oxide abatement 35
3.2.4 Parameters updates 35
3.2.4 Renewable energy mix 36
3.3 Comparison of mitigation scenarios and baseline 36
3.4 Inverse parameterisation of the climate model ACC2 37

# 1. Enhanced weathering module

## 1.1 Carbon dioxide removal

We considered two distinct processes that lead to carbon dioxide removal from enhanced weathering: the geochemical capture and the biotic capture. Here, we first describe the release of these elements as modelled in GET-ACC2, and then each of the capture processes.

### 1.1.1 Weathering rates

In the main text, we assume that the grain size is 20µm. The range of weathering rate used in this study is 1% to 26% per year. The low end is reported in Rinder and von Hagke (2021)[1]. The high end is the mean weathering rate in ORCHIDEE, where the values are based on Strefler et al. (2018)[2] and on local temperatures. For a temperature of 25°C and a pH of 5.85, Rinder and von Hagke report a weathering rate of 2.63 $10^{-12}$ mol.$g^{-1}$.$s^{-1}$, which is 12.7 times lower than what one obtains with equation G-2 in Strefler et al. (2018), with the same temperature and pH, notably because of a lower reactive surface. Given these diverse estimates of weathering rate, we assume that the uncertainty range of weathering rate is uniformly distributed between 1% and 26% per year. This uncertainty range is small compared to the 5-95% uncertainty range in Strefler et al. (2018)[2], which varies by a factor of a thousand. However, due to the critical role of weathering rate, we investigate the impacts of very low values separately. We also assume that all minerals contained in basalt are weathered simultaneously.

The weathering rates are strongly influenced by the rock types, the in situ physicochemical and hydrological conditions as well as by biological processes, which leads to a large dispersion of the experiments results. Furthermore, robust and standardised methods for measuring CDR and rock weathering are still lacking, which may also explain the dispersion of the observed weathering rates. In the following table, we reviewed the recent literature [1–15] on enhanced weathering experiments. The reported weathering rates were scaled to a value that would correspond to a grain size of 20µm. More precisely, if $w_r(d_1)$ is the weathering rate of a feedstock of grain size $d_1$, we have $\frac{w_r(d_1)}{w_r(d_2)} = (\frac{d_2}{d_1})^{\alpha}$. It is important to note that the relationship between weathering rate and grain size is uncertain, and that the existing literature reports both sublinear ($\alpha < 1$, e.g. ref[3]) and superlinear ($\alpha > 1$, e.g. ref[1,2]) relationships between weathering rates and the inverse of grain sizes. For simplicity, we assumed that weathering rates are proportionate to the inverse of grain sizes ($\alpha = 1$), as would be the case for perfect spheres, whose weathering rates depend on the reactive surface. For each mineral, we assume an exponential law of dissolution like in the main text, where the share of dissolved basalt $x_t$ follows: $1 - x_t = exp(-w_r t)$. For experiments during less than one year, we thus to obtain the share dissolved after 1 year as follows: $1 - x_T = exp(\frac{T}{D} ln(1 - x_D))$ where D is the duration of the equation and T is one year.

| Study type | Grain size and reported weathering rate (% per year) | Linearly scaled weathering rate for 20µm-grains | Rock type | Ref |
|---|---|---|---|---|
| Reactor simulating humid tropical soil. | size: 300µm<br>rate: 12-19 %/year | 173-283 %/year | Basaltic flow (“Arenal”) | Ryan et al 2024 |
| Reactor simulating humid tropical soil. | size: 47.2 µm<br>rate: 30-42 %/year | 70-100 %/year | Basaltic flow (“Barva”) | Ryan et al 2024 |

| | | | | |
|---|---|---|---|---|
| Reactor simulating humid tropical soil. | size: 14.1 µm<br>rate: 20-78 %/year | 14-55 %/year | Basalt flow ("BHVO-1") | Ryan et al 2024 |
| Reactor simulating humid tropical soil. | size < 45µm<br>rate: 3-27 %/year | 7-63 %/year | Basalt (BR-fine) | Vanderkloot et al 2023 |
| Reactor simulating humid tropical soil. | size <45µm<br>rate: 6-41 %/year | 14-96 %/year | Basalt (PV-fine) | Vanderkloot et al 2023 |
| Planted Mesocosm | size ~ 100µm<br>rate: 30 %/year (1.6 t/ha) | 120 %/year | Olivine | ten Berge et al. 2012 |
| Planted Mesocosm | size ~ 100µm<br>rate: 2 %/year (204 t/ha) | 10 %/year | Olivine | ten Berge et al. 2012 |
| Planted Mesocosm | size: 43 µm<br>rate: 0.03%/year | 0.06 %/year | Dunite | Amann et al. 2018 |
| Planted Mesocosm | size: 1020µm<br>rate: 0.01%/year | 0.5 %/year | Dunite | Amann et al. 2018 |
| Planted Mesocosm | size: 20µm<br>rate: 91%/year (10t/ha) | 91%/year | Olivine | Dietzen et al. 2018 |
| Planted Mesocosm | size: 20µm<br>rate: 40 %/year (50t/ha) | 40 %/year | Olivine | Dietzen et al. 2018 |
| Planted Mesocosm | size: 25 µm<br>rate: 94 %/year | 117 %/year | Wollastonite | Haque et al. 2019 |
| Planted Mesocosm | size: 1250µm<br>rate: 17 %/year | 600 %/year | Basalt | Kelland et al 2020 |
| Planted Mesocosm | size: 128µm<br>rate: 21.7 %/year | 139 %/year | Basalt | Kelland et al 2020 |
| Planted Mesocosm | size: 35µm<br>rate: 23 %/year | 40 %/year | Basalt | Reershemius et al 2023 |
| Field Trial (oil palm plantation) | No weathering measured | No weathering measured | Basalt | Larkin et al 2022 |
| Field trial (Potato field) | size ~ 10µm<br>rate: 93%/year | 41 %/year | Wollastonite | Haque et al 2020 |
| Field trial (Soybean field) | size ~ 10µm<br>rate: 43%/year | 21 %/year | Wollastonite | Haque et al 2020 |
| Field Trial (Corn plantation) | size: 367µm<br>rate: 3-6 %/year | 48-110 %/year | Basalt | Beerling et al 2024 |
| Field Trial (Rice paddy) | size < 75 µm<br>rate: 91%/year | 341%/year | Wollastonite | Wang et al 2024 |
| Soil core study | size: 125-250µm<br>rate: 0.04-0.05 %/year | 0.5-0.8 %/year | Basalt | Buckingham et al 2022 |

| | | | | |
|---|---|---|---|---|
| Modelling (reactive transport model) | size: 714µm<br>rate: 5.7 %/year | 205 %/year | Basalt (Oregon) | Lewis et al 2021 |
| Modelling (reactive transport model) | size: 1128µm<br>rate: 5.5 %/year | 308 %/year | Basalt (Craigmill) | Lewis et al 2021 |
| Modelling (reactive transport model) | size: 1531 µm<br>rate: 4.7 %/year | 362 %/year | Basalt (Tichum) | Lewis et al 2021 |
| Modelling (reactive transport model) | size: 267µm<br>rate: 0.8 %/year | 11 %/year | Basalt (Blue ridge) | Lewis et al 2021 |
| Modelling (reactive transport model) | size: 1767µm<br>rate: 3.8 %/year | 336 %/year | Basalt (Tawau) | Lewis et al 2021 |
| Modelling | size: 20µm<br>rate: 1.03 %/year | 1.03 %/year | Basalt | Rinder et von Hagke 2021 |
| Modelling | size: 20µm<br>rate: 19.6 %/year | 19.6%/year | Basalt | Strefler et al 2018 |

*Ryan et al, 2024:* The share of minerals present in the rocks that were leached after 14 days are given in their table 4b. For each rock type, we report the range of weathering rates of Ca and Mg scaled by the grain size.

*Vanderkloot et al 2023* reports a similar experimental design as Ryan et al, 2024. We do the same calculation to extrapolate annual weathering rates, considering only the cases with fine grain size (<45µm) to limit the errors when scaling to 20µm grains.

*Kelland et al 2020* reports that 17% and 21.7% of the maximal $CO_2$ removal were obtained after 1 year, for relatively large grains (p80 = 1350µm, p50 ≈ 700µm). The weathering rates for ten Berge et al. 2012, Amann et al 2018, Dietzen et al 2018 and Haque et al 2019 were obtained from the table 3 of Kelland et al 2020

*Reershemius et al 2023* reports that 15.7% of the maximum $CO_2$ removal was obtained after 235 days for grain size close to 20µm (p80 = 35µm, p50 ≈ 20µm).

*Larkin et al 2022* reports no difference of $CO_2$ drawdown through alkalinity export between basalt-amended and reference fields.

*Beerling et al 2024* reports a 16 ± 6% loss of cations from basalt applied on crop fields over 4 years. This corresponds to a mean weathering rate of around 4 ± 2% per year. Since the reported grain size in this study is 267 µm, and the weathering speed is proportional to the reactive surface of the grains, we can extrapolate that 20µm-sized grains should weather more than ten times faster.

*Haque et al 2020* reports that in one field, the application of wollastonite at the rate of 1.24t/ha resulted in a CDR of 0.32t$CO_2$/ha after 5 months, which means that around 70% of the rocks have been weathered in 5 months assuming a stoichiometric rate of 1 mole of $CO_2$ sequestered per mole of wollastonite weathered after full precipitation of carbonates. The same calculation for another field,

where coarser wollastonite was applied, indicates that around 20% of the initial input was weathered after 20 weeks.

Buckingham et al 2022 report the release rates (in mol.cm$^{-2}$.s$^{-1}$) of calcium and magnesium ions from basalt grains of size 150-250 µm. As they also report the mass composition of their basalt feedstock and its reactive surface, it is straightforward to derive the corresponding weathering rate in %/year. These results they report have been contested (ref[16], with a response in ref[17]).

Lewis et al 2021 simulate the weathering of different basalt feedstocks depending on their mineralogy using a reactive transport model. They report the share of maximum CDR after 15 years, which we assume to be equal to the share of rock weathered.

Rinder et von Hagke 2021 simulate the weathering of 20-µm sized grains and obtain lower weathering rates than Strefler et al 2018, due to a lower reactive surface.

Due to the importance of weathering rates, we reproduced Figures 2 and 3 for weathering rates equal to 1%/year and 25%/year, which are the bounds of the assumed uncertainty range.These figures show that although the biotic and geochemical CDR are higher at high weathering rates, the biotic effect is, relative to the geochemical effect, more efficient at low weathering rates.

#### 1.1.1.1 Weathering rate = 1% per year

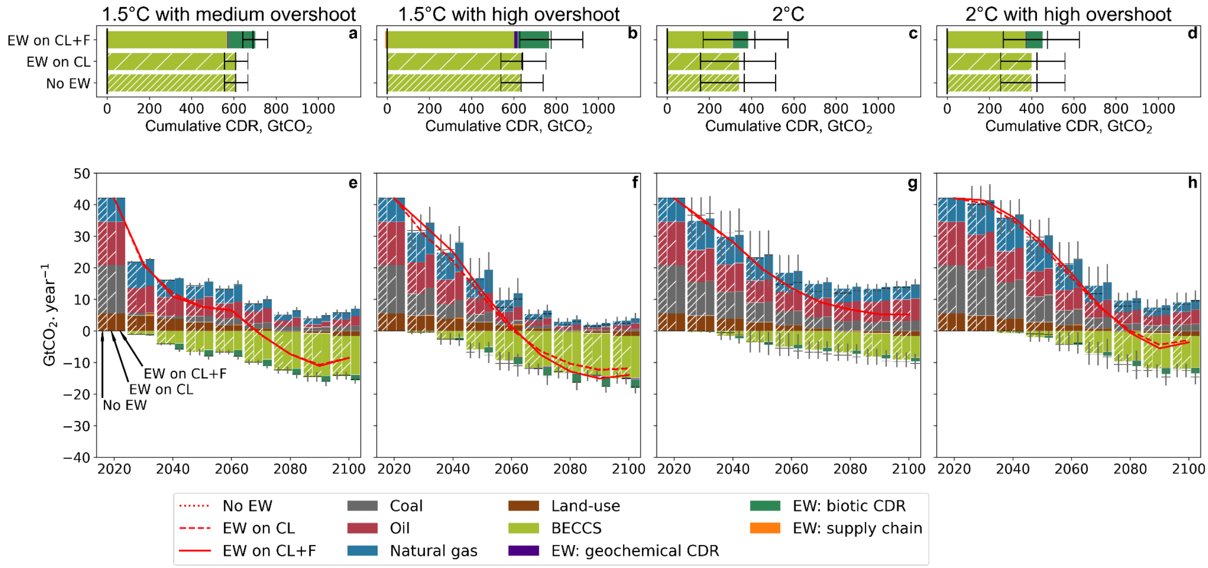


**Supplementary Figure 1| Carbon dioxide emissions from three CDR portfolios for different climate targets across the 21st century.** Discount rate = 5%. Weathering rate= 1% per year.

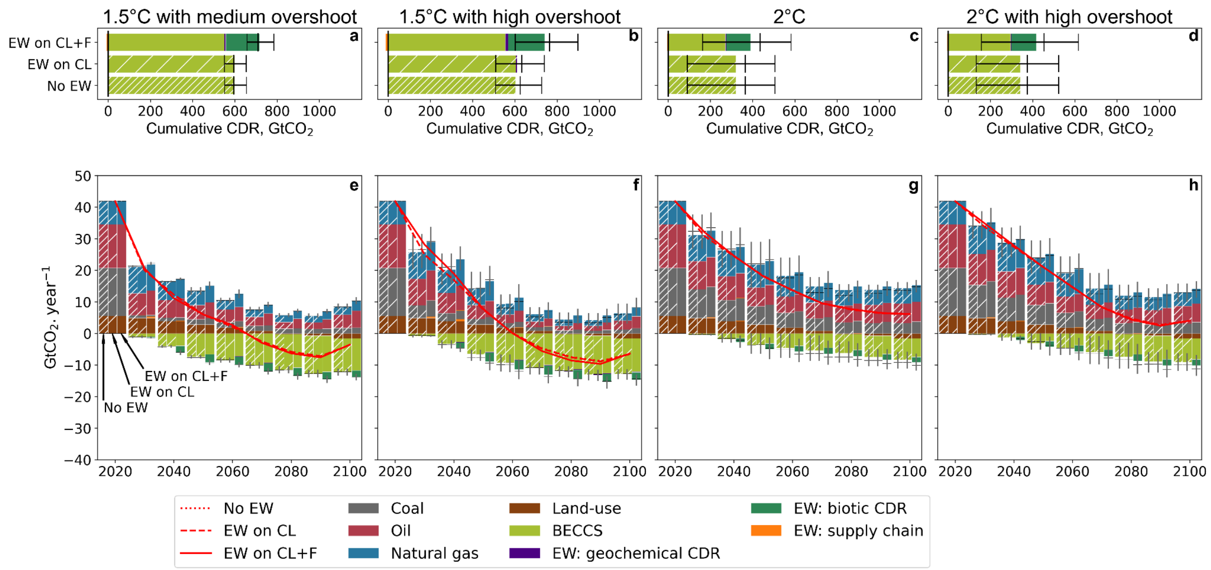


**Supplementary Figure 2| Carbon dioxide emissions from three CDR portfolios for different climate targets across the 21st century.** Discount rate = 2%. Weathering rate= 1% per year

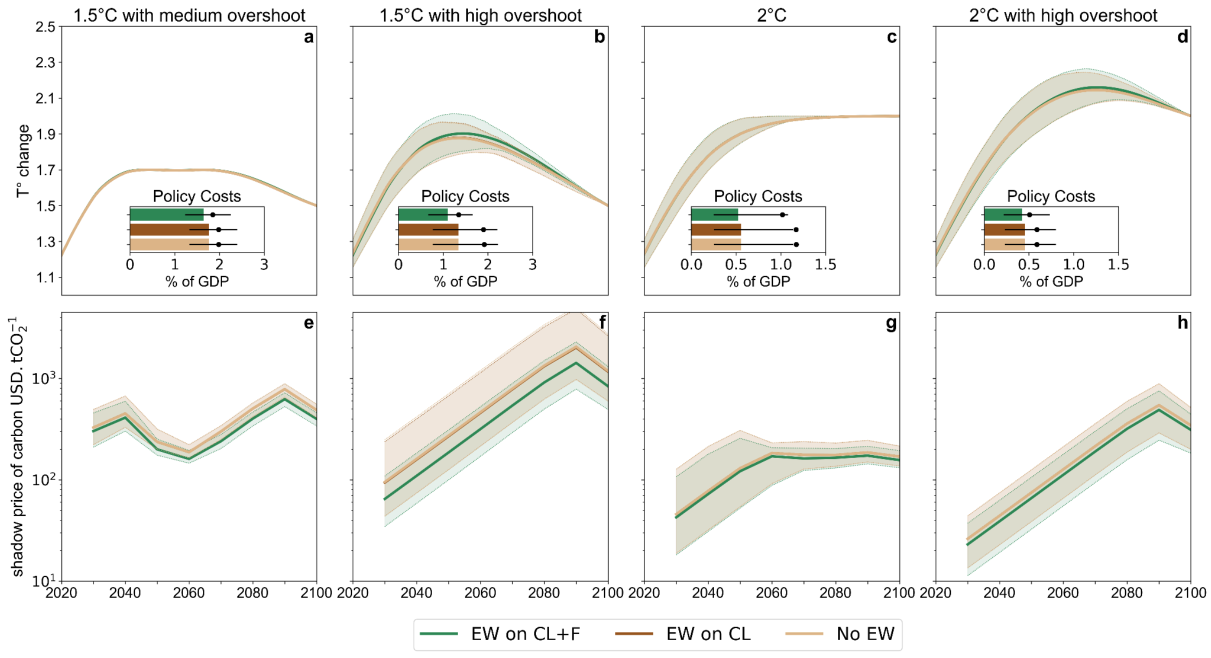


**Supplementary Figure 3|| Temperature, policy costs and carbon price pathways across the 21st century.** Discount rate = 5%. Weathering rate= 1% per year.

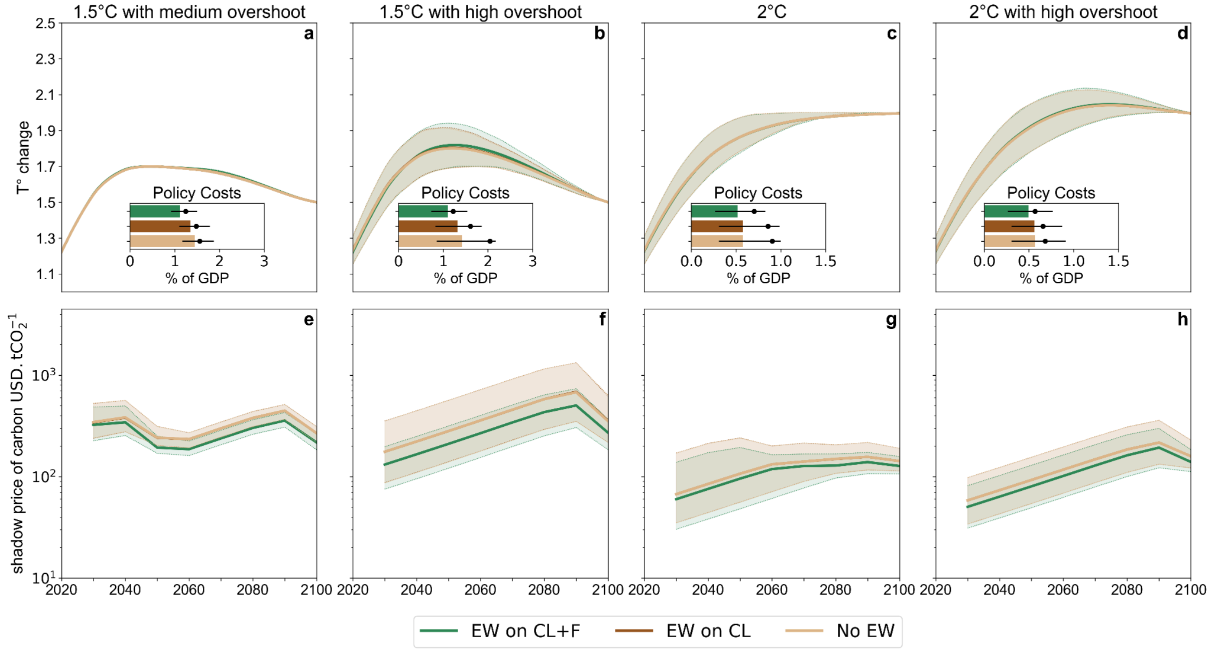


**Supplementary Figure 3| Temperature, policy costs and carbon price pathways across the 21st century.** Discount rate = 2%. Weathering rate= 1% per year.

#### 1.1.1.2 Weathering rate = 25% per year

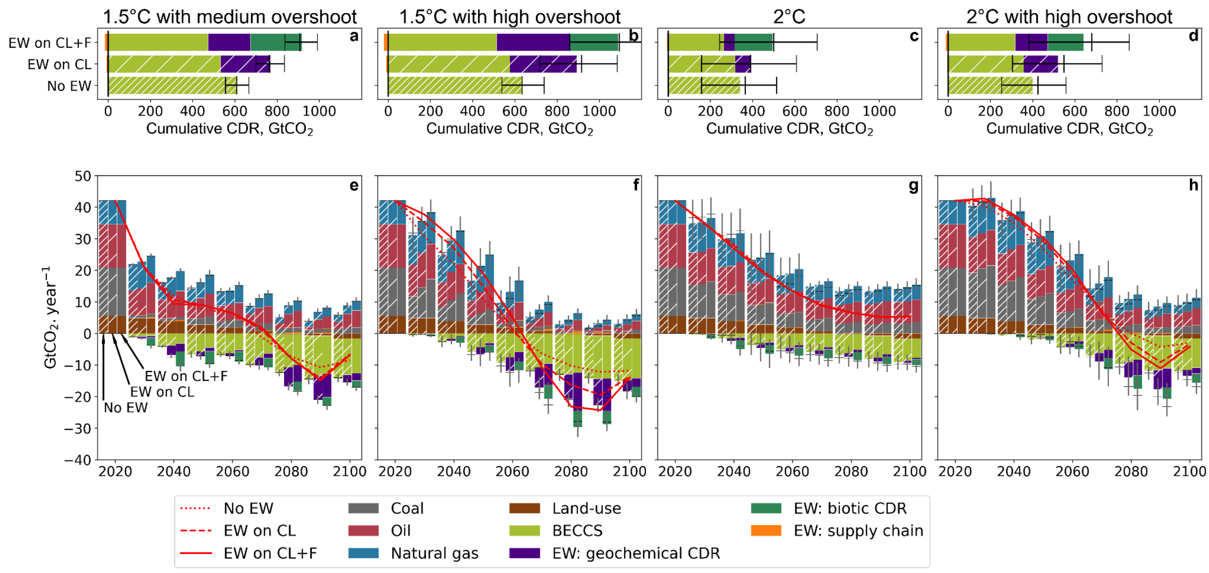


**Supplementary Figure 4| Carbon dioxide emissions from three CDR portfolios for different climate targets across the 21st century.** Discount rate = 5%. Weathering rate= 25% per year.

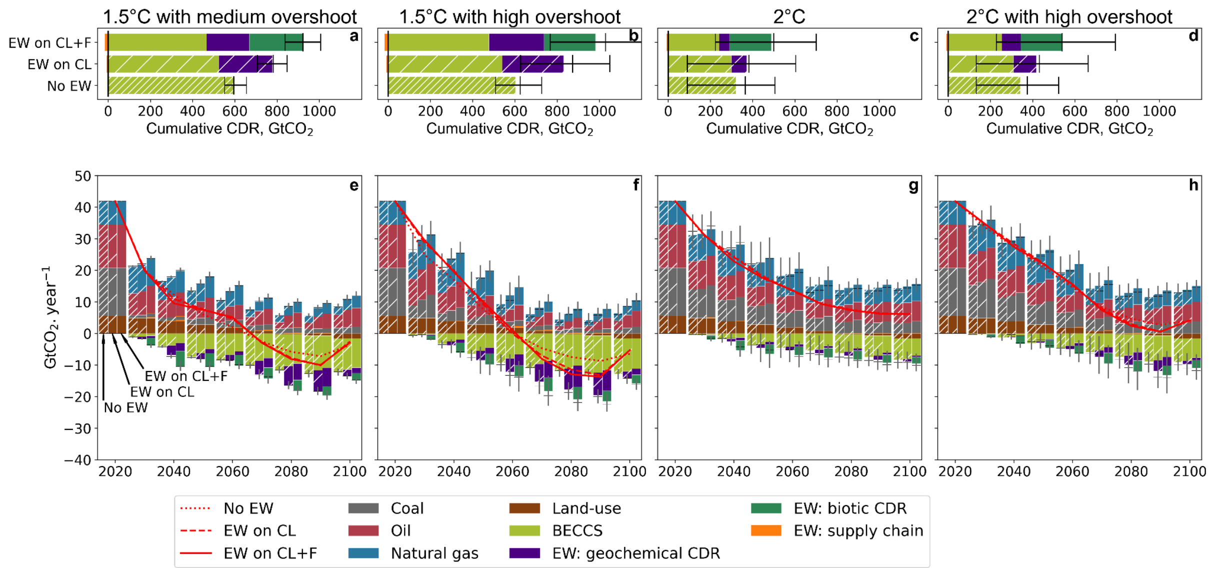


**Supplementary Figure 5| Carbon dioxide emissions from three CDR portfolios for different climate targets across the 21st century.** Discount rate = 2%. Weathering rate= 25% per year

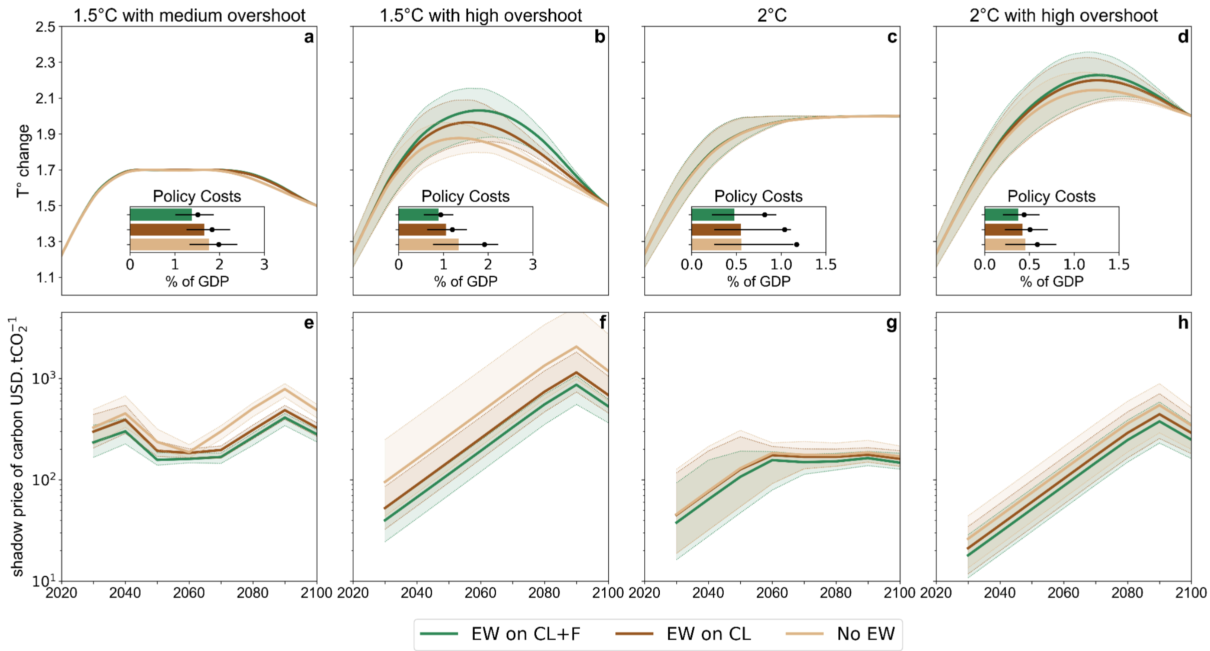


**Supplementary Figure 6| Temperature, policy costs and carbon price pathways across the 21st century.** Discount rate = 5%. Weathering rate= 25% per year.

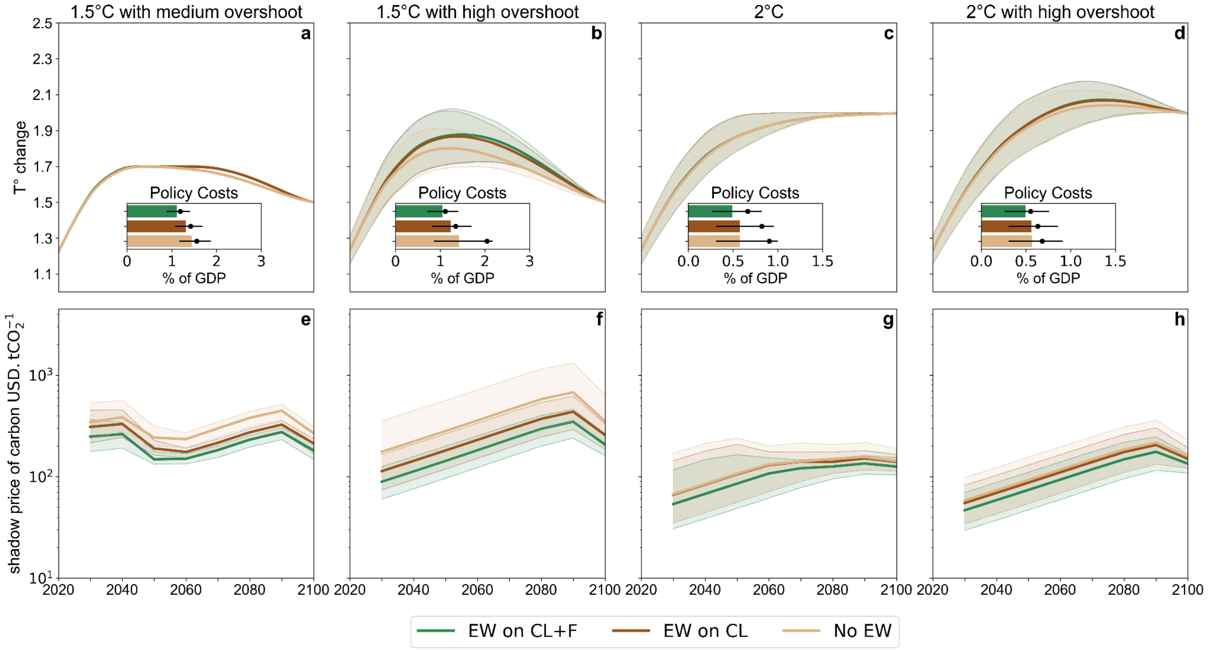


**Supplementary Figure 7| Temperature, policy costs and carbon price pathways across the 21st century.** Discount rate = 2%. Weathering rate= 25% per year.

### 1.1.2 Calibration of the model

The response of the net primary production (NPP) to basalt amendment in GET-ACC2 is calibrated using simulations from the ORCHIDEE-CNP land-surface model. Previous publications have described the phosphorus cycle in ORCHIDEE-CNP and the model validation [18,19].

The ORCHIDEE-CNP scenarios assume a uniform application of basalt dust over all land surfaces at the beginning of 2018, with different application rates: 0, 0.5, 1, 2, 3, 4, 5 $kg.m^{-2}$. The simulation runs until the end of 2200. The temperature, climate and atmospheric composition are unchanged from current levels until the end of 2200 in the simulation. The weathering rate only depends on temperature in these simulations. We use two kinds of output data for calibration: the net primary production, which is resolved over a 0.5° grid, at the plant functional type level, with a one-year timestep, and the geochemical $CO_2$ capture, at the same spatial and temporal resolution. The geochemical $CO_2$ capture follows an exponential decay, which is used to obtain the assumed weathering rate.

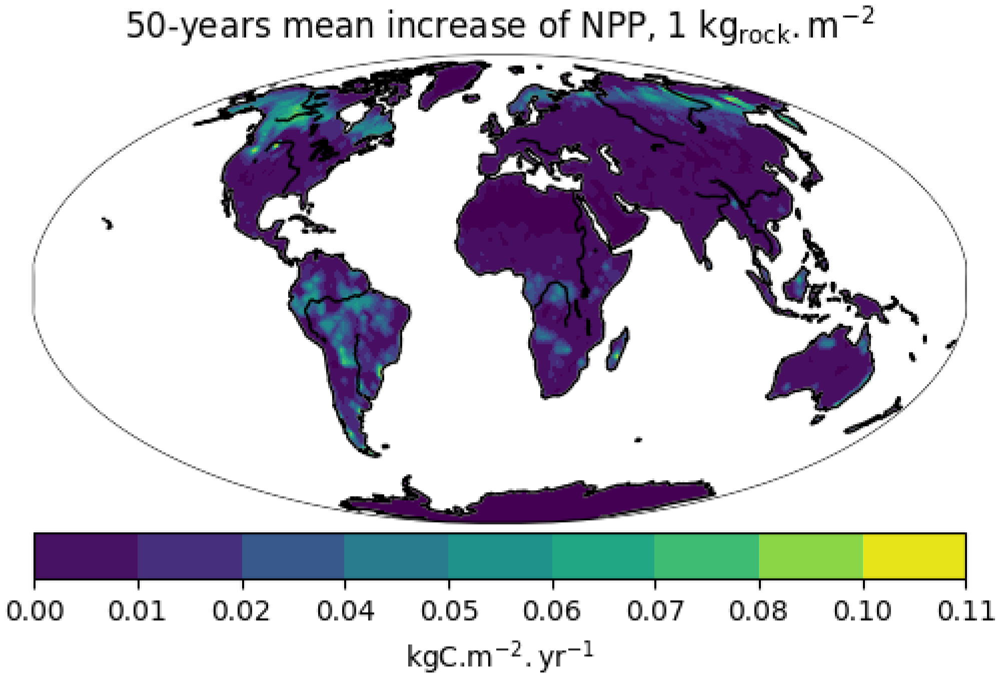


**Supplementary Figure 8|** Increase of the NPP, averaged over 50 years after the application of 1kg of basalt per $m^2$.

The NPP increase in forests following basalt application depends on local conditions[18]. It is stronger in tropical forests and in boreal forests. To make the most of the biotic carbon storage, it is more efficient to start applying basalt to the most "responsive" areas, that is to say the areas where the NPP increases the most after basalt application (Supplementary Figure 8). Therefore, the land surface pixels from ORCHIDEE-CNP are sorted from the most responsive to the least responsive to basalt application, and are grouped into M land response $classes_1$, $c_2$,...$c_M$. $c_1$ gathers the most responsive application sites, and $c_M$ the areas where the NPP increase following basalt application is the lowest. The classes are made to contribute equally to the total NPP increase (in GtC/year). Therefore, the surfaces of the classes $c_i$ increase with i, as a larger area is required to obtain a given NPP increase when basalt is applied on less responsive areas. It should be noted that the classes are not composed of contiguous pixels.

In GET-ACC2, at each given timestep, the basalt application is distributed across classes, and the application rate is uniform within each class. The costs of application in $\$/t_{rock}$ does not depend on the class, but is lower for higher mean application rates (in $kg/m^2$) across classes, incentivising higher basalt application rates in a lower number of classes. The more land response classes there are, the higher the possibility for efficient application of basalt is, but the more complex to solve the model is. We took a number of classes M=5 in the following, to limit the complexity of the model due to the large number of simulations (**Supplementary Figure 9**).

The phosphorus cycle emulator is built based on the following three assumptions: first, that the increase of net primary productivity is a function of the increase of soil phosphorus concentration. Second, the rate of increase of the soil phosphorus concentration is proportionate to the weathering of basalt. Third, we assume that the cycle of added phosphorus is open, with two phosphorus pools, the first one that is available to plants and which is fed by basalt weathering, the second one that is unavailable to plants and which exchanges phosphorus with the first one, but from which phosphorus also leaches away. The exchange rates follow classical diffusion dynamics.

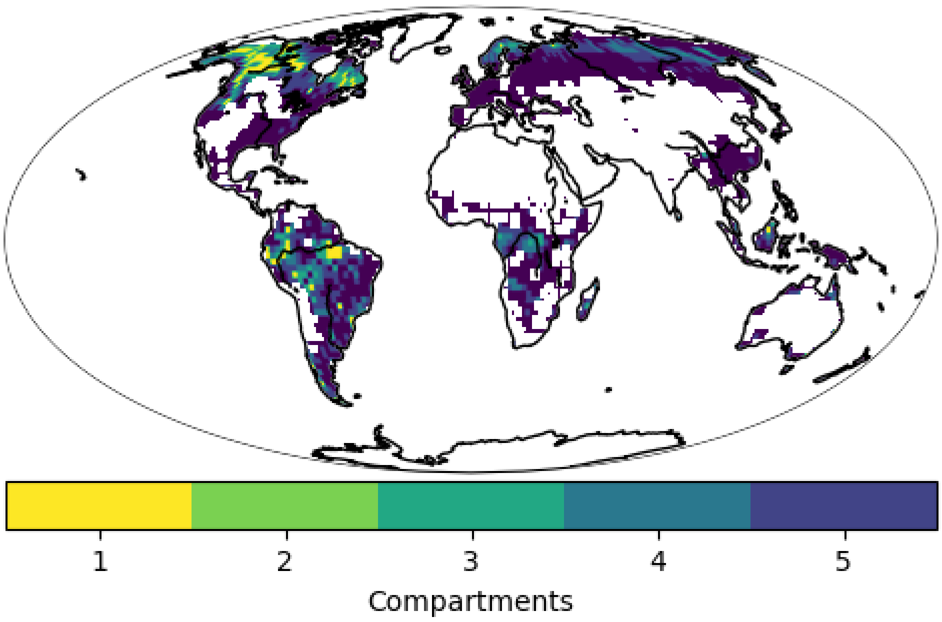


**Supplementary Figure 9|** Land response classes used in the current setting.

The increase of NPP in the land response class $i$, $\delta NPP_i$ (in GtC/year), increases as basalt weathers and releases phosphorus, and then decreases with time. The quantity of phosphorus released is proportionate to the amount of basalt applied. The increase of NPP increases with the quantity of added phosphorus $\delta c_{P,i}$, up to a maximum that depends on the class, following an exponential saturation pattern (E.5).

$$\delta NPP_i = \delta NPP_{i,max}\left(1 - e^{-\alpha_i \delta c_{P,i}}\right) \quad \text{(E.5)}$$

The dynamic evolution of the soil phosphorus concentration $\delta c_P$ is designed to reproduce the results of ORCHIDEE. It is modelled with an auxiliary pool of phosphorus which is unavailable to plants. It exchanges phosphorus with the soil concentration with exchange rates $1/\tau_{p,i}$ (from the soil solution phosphorus to the unavailable phosphorus pool) and $1/\tau_{u,i}$ (from the unavailable phosphorus pool to the soil solution), and leaches with a decay time $\tau_{l,i}$ (**Supplementary Figure 10).**

$$\frac{d\delta c_P}{dt} = \frac{\lambda w_r B}{a_B} - \frac{\delta c_P}{\tau_p} + \frac{\delta u_P}{\tau_u} \quad \text{(E.6a)}$$

$$\frac{du_P}{dt} = \frac{\delta c_P}{\tau_p} - \frac{\delta u_P}{\tau_u} - \frac{\delta u_P}{\tau_l} \quad \text{(E.6b)}$$

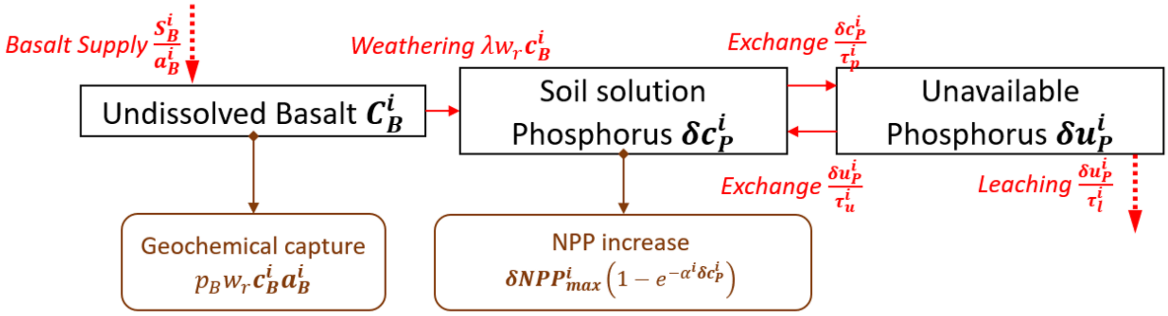


**Supplementary Figure 10 | Phosphorus cycle emulation in land response class *i*.** Rectangles: phosphorus pools. Red arrows: phosphorus flows. Brown arrows: effects of enhanced weathering on the carbon cycle.

For each land response class $i$, the parameters $\alpha_i$, $\delta NPP_{i,max}$, $\tau_{p,i}$, $\tau_{u,i}$ and $\tau_{l,i}$ are calibrated on the ORCHIDEE-CNP simulations using a gradient descent procedure. For each box, two methods are used: a step by step calibration, and a direct calibration.

In the step by step procedure, the temporal signal is filtered to remove the noise. Then, $\alpha_i$ and $\delta NPP_{i,max}$ are determined by comparing the maximum NPP increase for different basalt application levels. $\delta NPP_i = \delta NPP_{i,max}\left(1 - e^{-\alpha_i \delta c_{P,i}}\right)$. To calibrated the remaining parameters $\tau_{p,i}$, $\tau_{u,i}$ and $\tau_{l,i}$, we follow a simple gradient descent procedure, using the python method scipy.optimize.minimize [20]. To compute the error function to minimise over a set of possible values of parameters, we solve the set of equations E6a, E6b with current parameters values, then compute the resulting NPP increase (E5) with the known values of $\alpha_i$, $\delta NPP_{i,max}$, and finally compute the quadratic difference with the filtered signal. In the direct calibration procedure, we directly calibrate $\alpha_i$, $\delta NPP_{i,max}$, $\tau_{p,i}$, $\tau_{u,i}$ and $\tau_{l,i}$ on the filtered signal using the gradient descent. For each land response class, we export the set of parameters that yield the smallest quadratic error with the unfiltered ORCHIDEE-CNP output.

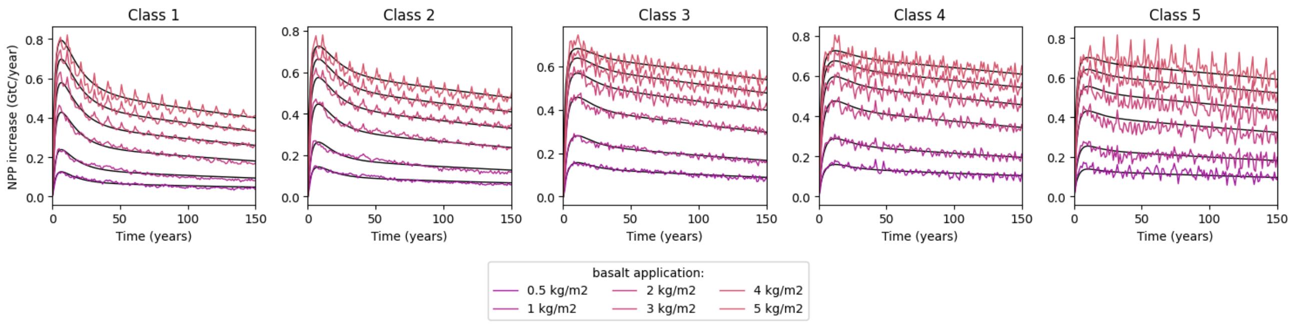


**Supplementary Figure 11 | Evolution of the net primary production (NPP) change**, for increasing basalt application rates. Basalt is applied at year zero. The y-axis is the increase of NPP compared to the baseline.

Evolution of the net primary production (NPP) change, for increasing basalt application rates. Basalt is applied at year zero. The y-axis is the increase of NPP compared to the baseline.

### 1.1.3 Long-term dynamics of the model

When the phosphorus addition has stopped, $\delta NPP^i$ gradually turns to zero, with a characteristic timescale of $\tau_l^i$. As a consequence, the additional carbon stored progressively returns to the atmosphere. (**Supplementary Figure 12, Supplementary Figure 13).**

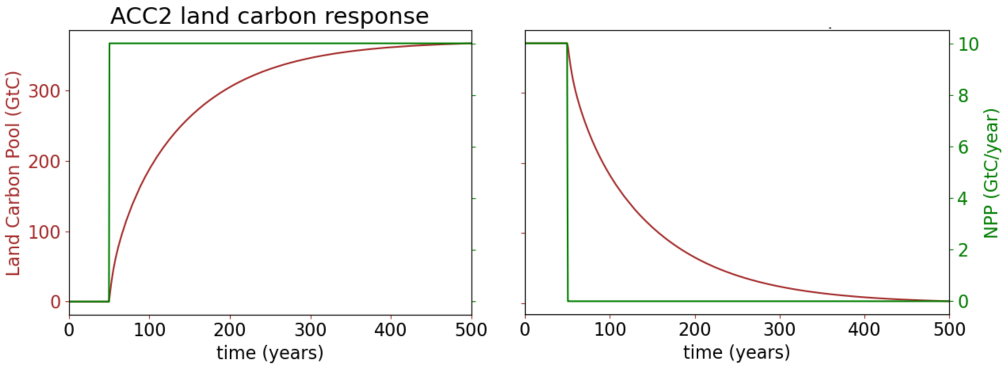


**Supplementary Figure 12 | Response of the land carbon pool to a NPP change from equilibrium** ($CO_2$ and temperature conditions of the year 2000).

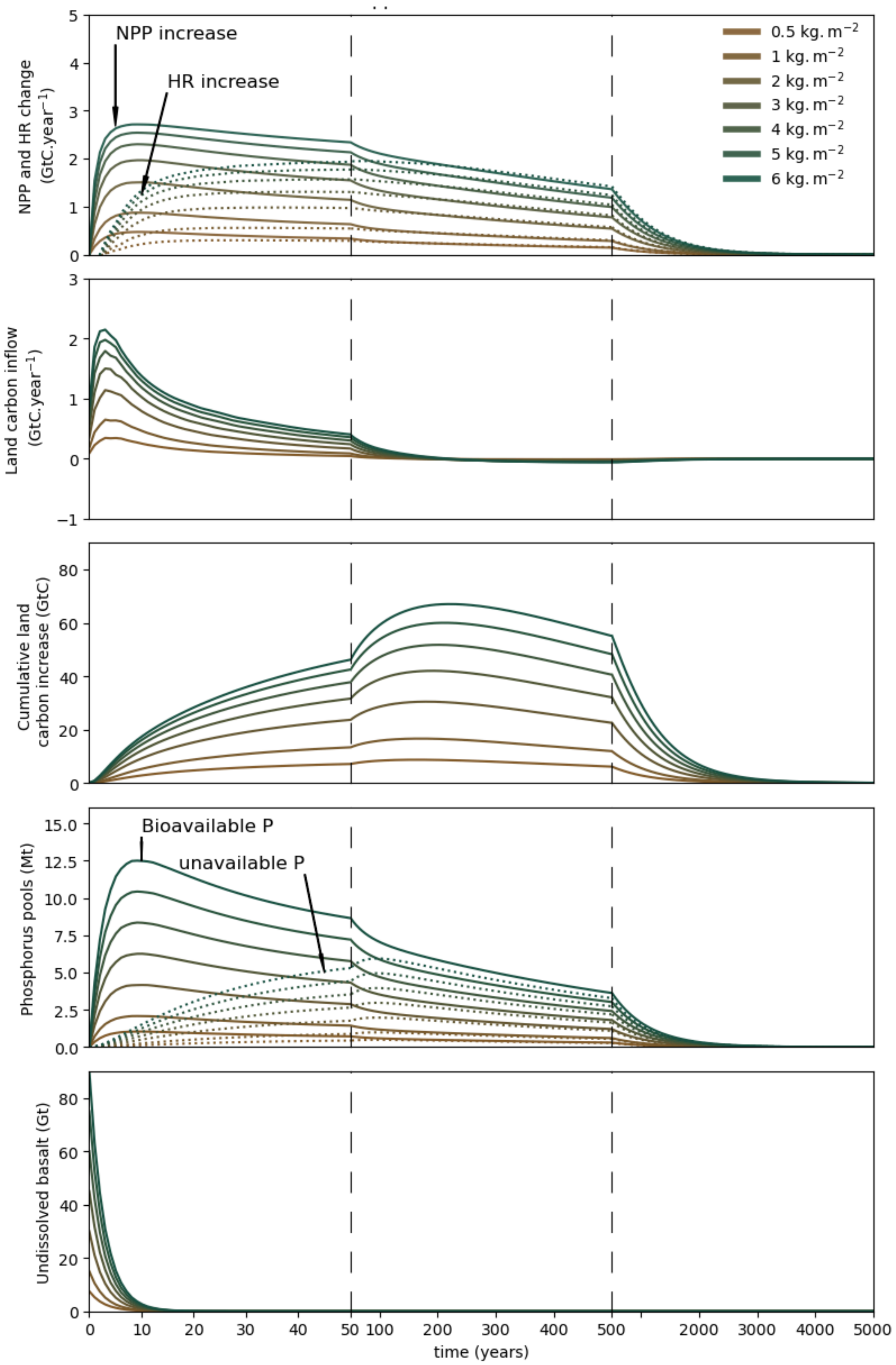


**Supplementary Figure 13 | Response of the land carbon stocks, land carbon flows and phosphorus pools** to a basalt addition at year 0. ( weathering rate = 25%), $CO_2$ and temperature conditions of the year 2000. **First panel:** dotted lines represent the increase of heterotrophic respiration (HR), solid line the increase of net primary production (NPP). **Second panel:** Net flow from atmosphere to land carbon (NPP-HR). **Third panel:** Cumulative land carbon increase.**Fourth panel:** dotted lines represent the unavailable phosphorus pool, solid line the soil phosphorus pool.

### 1.1.4 Spatial distribution of basalt application

Land response classes are built as follows: first, pixels are sorted out depending on their local increase in NPP after basalt application, from the most responsive to the least. Then they are grouped in N classes so that each class represents an equal share of the total NPP increase: the first class is therefore much smaller than the last one. Basalt is generally applied successively in the different classes, because it is cheaper to apply large concentrations of rocks and because carbon is sequestered during a long period after basalt application. The smallest class is therefore used first, then the second, etc. The fifth class is used only in the largest application case, the 1.5°C case with high OS (**Supplementary Figure 14**).

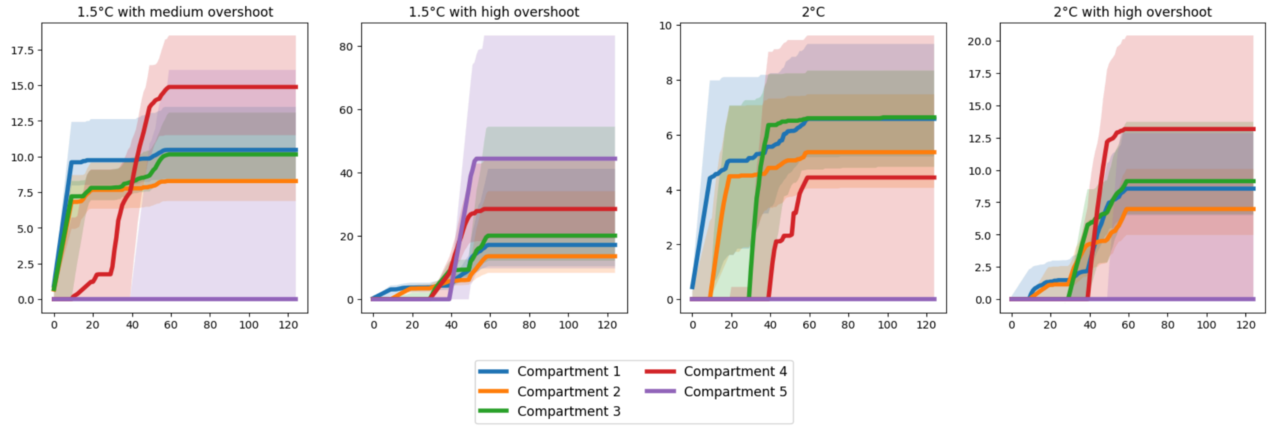


**Supplementary Figure 14| Cumulative basalt application in the different land response classes across the 21st century**. The solid line is the median, the shaded area is the 25-75% range.

## 1.2 Basalt supply

The basalt dust supply is integrated in GET7.1 with a 10 year timestep: costs and energy requirements of basalt supply are fed back into GET. Basalt must be mined, crushed and ground, and transported. Each of these steps requires energy. We assume that diesel or gasoline are used for mining and crop-fields spreading. Basalt could be transported by trains, trucks or ships. Here we assume that a minimum share of 70-90% must be transported by trucks. The energy requirements of transport increase the demand in each transport subsector (road, train or water freight). Transport modes are substitutable but a minimum share (70-90%) must be transported on the road.

| Process | Energy Intensity | Financial cost | Energy type | Demand in GET-ACC2 |
|---|---|---|---|---|
| Mining[21] | 0.02 EJ/Gt | - | Diesel | Diesel or gasoline |
| Mining and Crushing[2] | 0.02 EJ/Gt | 27.3$/t | Electricity | Electricity |
| Grinding[2] | mean: 0.2, range 0.07-0.6 EJ/Gt | included in crushing costs | Electricity | Electricity |
| Road Transport[22] | 0.0013 EJ/Gt/km | 0.08$/t/km | Endogenous | Road freight |
| Sea and rail transport[22] | 0.0002 EJ/Gt/km | 0.05$/t/km | Endogenous | Sea or rail freight |

| | | | | |
|---|---|---|---|---|
| Spreading (tractors)[23] | 0.078 EJ/Gt | 10.9$/t (ref.[2]) | Diesel | Diesel or gasoline |
| Spreading (aeroplanes) | 1.8-2.5 EJ/Gt | capacity cost : 170-214$/t/year O&M costs: 70-110$/t | Endogenous (kerosene, hydrogen or methanol) | Aviation fuel |

## 1.2.1 Transport distances

There are 3 cases : basalt is either spread on croplands with tractors, or basalt is spread on forest areas with tractors, or basalt is spread on forest areas with aircrafts.

*Application on croplands:* the transport distance for cropland application is derived from Strefler, et al (2018)[2]. The transport distance is generally lower than 200km. It starts from zero and increases (up to a maximum of 490 km) as long as the unweathered quantity of basalt applied on croplands increases, as we follow their assumption that the application rate is 15kg/m$^2$ which can be easily incorporated into soils.

*Application on forests:* we assume that the share of roadless forests (i.e. forest areas inaccessible by roads) is approximately the same as the share of global roadless areas, that is to say 80%[24]. This share can be expected to decline in the future, despite the threats roads pose to ecosystems[24]. On the other hand, applying basalt on forests by means of land transport can be challenging, even when a road is available. We therefore assume that between 70% to 90% of basalt application on forests must be spread with aircrafts until 2100. This share depends on the development of roads and on the share of forests that tractors can penetrate. Aerial application is likely to be more expensive and energy intensive than land-based alternatives. If the share of aircraft use were lower, the energy consumption would also be lower, and basalt application could become higher in a cost-optimisation model. Assuming a large part of aerial spraying is therefore pessimistic as far as costs are concerned.

We assume that the rock is ground at a location proximate to the mine. The mean straight-line distance between the airport and the closest area where basalt is available is 250 to 400 km (**Supplementary Figure 15)**. We estimate the average road distance to be around 350 to 560 km by assuming that the actual travel distance on the road is on average larger than the straight-line distance by a factor of the square root of 2 (i.e., a simple analogy from geometry). If basalt is applied with tractors, the distance between basalt source and application site is generally lower than 250 km (**Supplementary Figure 16)**, thus the road distance is estimated to be around 350 km under the assumption of the square root of 2. We therefore consider a range of 350 to 550 km for basalt transport distance.

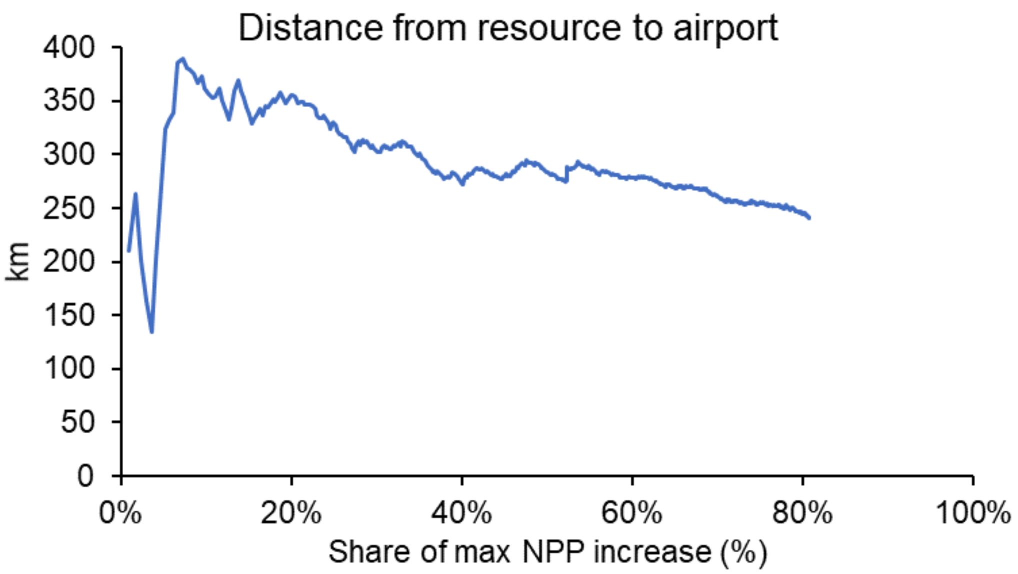


**Supplementary Figure 15| Straight line distance from basalt resource to the nearest airport from an application site.** The x-axis is the cumulative contribution from the application sites, ranked from the most reactive to the least reactive to basalt amendment, to the total NPP increase. The y-axis is the mean distance corresponding to these cumulative contributions. This image was created by the authors using the application sites from ORCHIDEE-CNP simulations, The distances are computed using the software QGIS. The database for basalt resources is the one used in Strefler, et al. (2018), GLiM[25]. The database for airports is the one used by Goll, et al. (2021)[18] openflights.org.

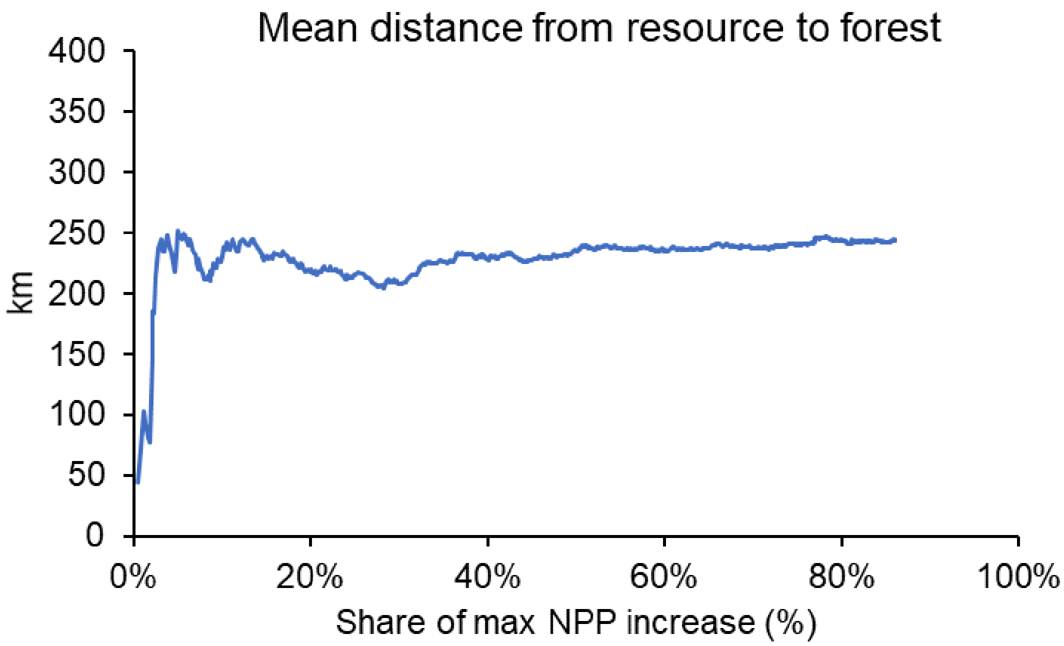

**Supplementary Figure 16 | Distance from basalt resource to application site** (straight line).The x-axis is the cumulative contribution from the application sites, ranked from the most reactive to the least reactive to basalt amendment, to the total NPP increase. The y-axis is the mean distance corresponding to these cumulative contributions. This image was created by the authors using the application sites from ORCHIDEE-CNP simulations, The distances are computed using the software QGIS. The database for basalt resources is the one used in Strefler, et al. (2018), GLiM[25]. The database for airports is the one used by Goll, et al. (2021)[18] openflights.org.

### 1.2.2 Cost of aircraft application

Our analysis aims to identify a range of energy requirements and financial costs for airborne spreading of basalt. A small agricultural aircraft like the AirTractor 802, which can be equipped with a dust spreader, could be used to spread basalt dust. This kind of aircraft is commonly used to spread limestone [26–28] although issues with rock discharge have been reported, due to the large particle size distribution[26]. The details of rock dust discharge are beyond the scope of this analysis, and more research would be needed on how to spread large quantities of basalt dust by air.

The following table summarises the key values used for our cost estimate:

| Feature | Source |
|---|---|
| Useful load : 4.3t | Guide to Air Tractor Aircraft |
| Cost: USD 1.8 million | https://air.one/ |
| Fuel consumption: up to 330 l/h | Guide to Air Tractor Aircraft |
| ground O&M costs: 120-210$/hour | Ref.[29] |
| Housing & Insurance: 5% of purchase cost each year | Ref.[29] |
| | |
| Pilot labour cost: 300$/hour | Ref.[29] |
| Speed: 306 km/h | AIR TRACTOR AT-802/802A Pilot Training Program |

The distance between the centre of spreading sites and the nearest airport ranges between 80 and 120 km (**Supplementary Figure 17**). We therefore assume a flying distance ranges between 160 and 240 km. It represents a flight duration of 31-47 minutes. Adding 10 minutes for spreading, the average flight duration would reach 41-57 minutes. This represents 226-314 litres of kerosene per

mission, or 7.6-10.6 GJ per mission, or 1.8 to 2.5 GJ/t of basalt applied. Assuming that 20 minutes are required to refuel and refill with basalt between each flight, and that the aircraft operates 10 hours per day, and that it is used 5 days out of 7, an aircraft can realise between 2030 and 2550 missions per year. Therefore, the capacity cost per ton/year is 170-214$/t/year (it depends on the average distance flown). We assume a lifetime of 30 years.

The hourly costs, including ground operations & maintenance costs, and labour, amount to 420-510 $/hour. With the previous assumptions, it takes 9 to 13 minutes of flight to spread 1 ton of rocks. Therefore, the total O&M costs per ton, including pilot's labour cost is 70-120$ per ton, or 56-102$ if the discharge takes 1 minute instead of 10. Finally, maintaining the landing trail, housing the plane and insuring it costs around 5% of the aircraft purchase cost each year. We are considering only existing landing trails.

The cost depends on the number of hours flown. A very low application rate (in kg per $m^2$) would result in a longer application duration, and therefore in higher costs per ton applied. We assume that the energy and financial costs of aerial application are proportionate to the duration of each flight. The flight duration is equal to: $D_{tot} = D_{travel} + \frac{m}{vLr}$ where $D_{travel}$ is the time required to access (and return from) the spreading site (31-47 minutes), m is the load of basalt carried (4 tons), v is the velocity (306 km/h), L is the spreading width (assumed to be 10 m), and r is the application rate (in $kg/m^2$). If the discharge takes 10 minutes, the application rate equals to 0.008 kg $/m^2$. If the discharge takes 1 minute, the resulting application rate is 0.08kg/$m^2$.

The use of electric drones could also be considered and be cheaper. However, considering their current performances, they cannot carry large amounts of rocks up to the most remote areas, and the application over forests with drones could therefore require developing more roads. Therefore, we consider the application by aircrafts only.

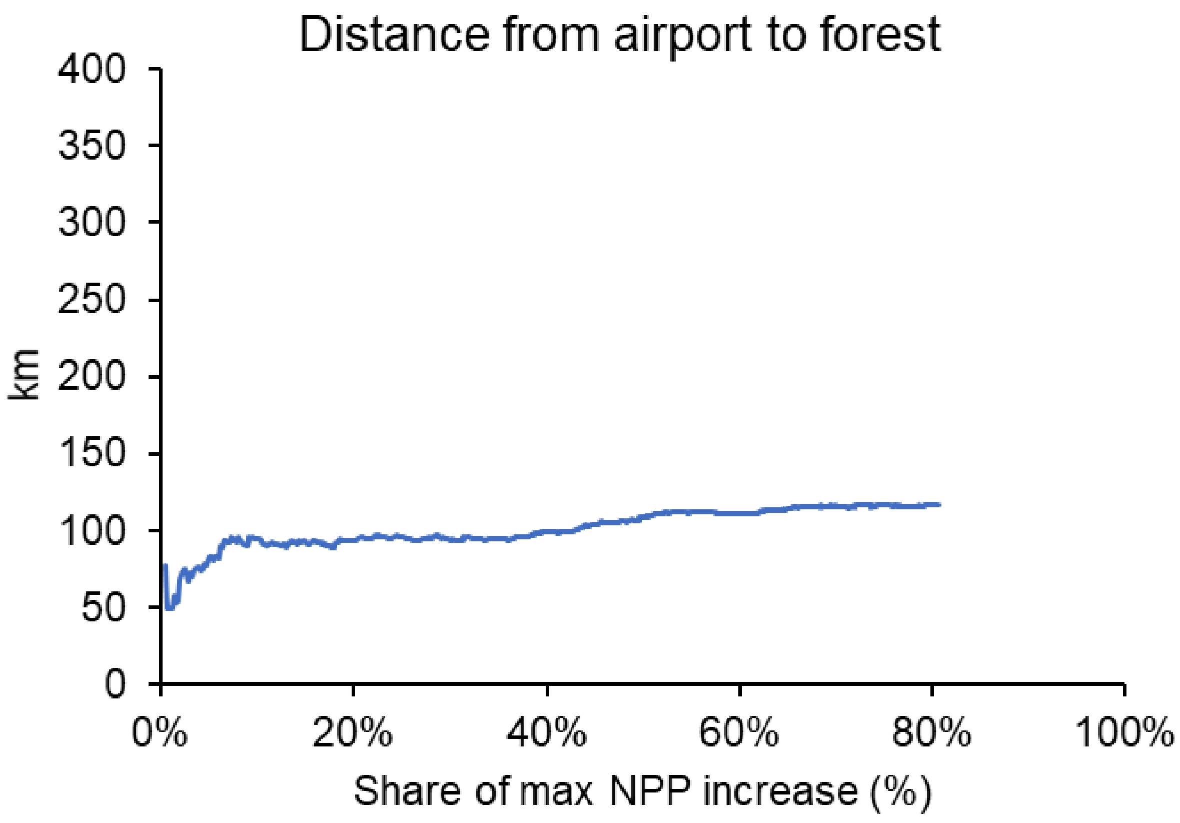

**Supplementary Figure 17 | Distance between application site and nearest airport.** The x-axis is the cumulative contribution from the application sites, ranked from the most reactive to the least reactive to basalt amendment, to the total NPP increase. The y-axis is the mean distance corresponding to these cumulative contributions. This image was created by the authors using the application sites from ORCHIDEE-CNP simulations, The distances are computed using the software QGIS. The database for basalt resources is the one used in Strefler, et al. (2018), GLiM[25]. The database for airports is the one used by Goll, et al. (2021)[18] openflights.org.

## 1.3 Break-even $CO_2$ price of basalt application

Basalt is applied when the net present value of costs becomes lower than that of benefits, which are defined as the discounted sum of future CDR multiplied by future carbon prices. Intertemporal effects, especially biotic CDR, are crucial due to CDR continuing for decades after basalt dust application. It is important to note that although we analyse the costs and benefits of basalt application, GET-ACC2 optimises the deployment of enhanced weathering without resorting to intermediate prices and benefits computations. The dual solution of the optimization offers a means to retrospectively determine the marginal energy, non-energy costs and the benefits of basalt application on both croplands and forests (**Supplementary Figure 1.3.5**).

### 1.3.1 Costs

The cost of basalt application can be broken down into two categories: non-energy cost and energy cost. The non-energy cost has a constant part and a variable part which depends on the quantity of basalt applied. The energy cost is determined by aggregating the quantity of all energy carriers used, each multiplied by their respective energy prices which vary depending on the scenario.

**Non-energy costs**

*Production:* the production cost of basalt dust is $27 per ton.

*Transportation:* when applied on croplands, transportation costs rise as more distant fields are accessed for increased basalt application, reaching a maximum of $34 per ton. An additional $11 per ton is incurred for spreading. Hence, non-energy costs escalate from $38 to $72 per ton applied as (endogenous) application rates increase. If basalt is applied in forests, transportation costs range from $25 to $37 per ton, depending on the proportion applied with tractors (10-30%) and the distance between the mine and airport (350-550 km).

*Application:* aerial spreading operation costs (excluding energy expenses) vary based on ground operation and maintenance costs, as well as the endogenous application rate (costs approach infinity as application rates decrease towards zero kg per $m^2$), ranging from $56 to $102 per ton for rapid discharges, depending on flight duration to the nearest airport. Pilot fees constitute approximately 60% of this cost. With an aircraft lifespan of 30 years and a 5% discount rate, the annual cost for spreading

capacity priced at $170-210 per ton per year is $11-13.7 per ton, to which we add 5% of the purchase cost for housing and insurance costs, $8.5-10.5 per ton. Thus, non-energy costs for aerial spreading range between $84 and $123 per ton. Considering the proportion of basalt applied with tractors, direct forest spreading costs range from $55 to $104 per ton. Incorporating basalt extraction, grinding, and transport, the total non-energy cost per ton applied per aircraft in forests is $136-191. This cost escalates with increased (exogenous) aerial application share, (exogenous) average distance between airports and application sites, and (exogenous) ground operation and maintenance expenses. Including the 10-30% applied with tractors, the total range of non-energy cost of forest application is $114-179 per ton.

**Energy costs**

*Production:* the energy cost for basalt dust production is minimal, primarily utilising electricity at an average rate of 0.2 GJ/ton (ranging from 0.07-0.6 GJ/t). In the GET-ACC2 model, electricity prices remain relatively stable over time in the business-as-usual scenario (around $14/GJ or $50/MWh). Prices increase in climate mitigation scenarios, particularly in the first half of the century but rarely surpass $28/GJ or $100/MWh, except in the 1.5°C case with medium overshoot, necessitating rapid decarbonization of the electric system. Thus, electricity costs typically range from $2.8 to $5.6 per ton, with uncertainty on basalt grinding electricity intensity leading to a range of $1-17 per ton.

*Transport:* the energy cost per ton-km for basalt transport varies from $0.02/t/km in the business-as-usual scenario (where no basalt is applied) to approximately $0.06-0.1/t/km in climate mitigation scenarios. This cost correlates positively with the carbon price and depends on the proportion of basalt transported by roads. Consequently, transporting basalt incurs an energy cost of up to $40 per ton for cropland application and $20-55 per ton for forest application.

*Application:* the energy consumption for airborne spreading ranges from 1.8-2.5 GJ/t, whereas for terrestrial spreading, it is 0.078 GJ/t. Since kerosene and diesel are priced equally in the model, the energy cost for airborne spreading is 23 to 32 times higher than that for terrestrial spreading. The cost of kerosene and diesel follows an affine function of the carbon price. When carbon prices exceed approximately $300/tCO2, the cost of kerosene surpasses that of hydrogen (around $40/GJ), leading to its substitution by hydrogen as aviation fuel, limiting further increases in aviation fuel prices. Consequently, aviation fuel prices range from $8 to $50 per GJ primarily depending on the carbon price, resulting in energy costs for basalt airborne spreading between $15 and $125 per ton. However, if carbon prices are very high in the near-term, technological diffusion constraints hinder the quick substitution of kerosene by hydrogen, and energy costs can exceed $125 per ton. This is the case in low-overshoot cases if the climate sensitivity is high. The energy cost of terrestrial spreading remains below $4 per ton. Overall, energy costs for cropland application range from $5 to $60 per ton. These costs primarily hinge on the quantity of basalt applied, endogenous carbon prices, and the exogenous energy intensity of basalt comminution. In contrast, energy costs for airborne forest application are significantly higher, spanning from $36 to $200 per ton, with a strong sensitivity to carbon prices. Since 10-30% share of basalt is applied with tractors, the mean energy costs of forest amendment with basalt dust range from $32 to $185 per ton.

Adding the energy and non-energy costs, the total costs of EW on croplands range from $43 to $132 per ton, and the cost of the first ton applied ranges from $43 to $58. The total costs of enhanced weathering on forests range from $146 to $364 per ton, in line with a previous assessment assessment (162-325$/t, see ref[30]), and the share of energy costs within total costs is primarily driven by the carbon price (**Supplementary Figure 18**), besides exogenous parameters variation.

Our results align with the few existing global-scale studies on EW application, which have so far been limited to the case of cropland application[2,31–33]. Ref[2,32] reports a global annual potential of 4 $GtCO_2$/year, limited by suitable croplands area, at a cost of 200$/$tCO_2$. Notably, the EW module in ref[31] as well as our cropland application module have been designed following ref[2,32], and we obtain similar CDR magnitude and application costs. Ref[33] estimated a median technical potential of 3.2$GtCO_2$/year (uncertainty range of 2-5 $GtCO_2$/year) under 2°C scenario, considering application only in China, India, United States, Canada, Indonesia, Mexico, Brazil, France, Germany, Italy, Spain and Poland, and reports country-specific median costs ranging from 60 to 420$/$tCO_2$ depending on the country. This larger cost range reflects their bottom-up approach, which does not assume a globally uniform marginal carbon price.

### 1.3.2 Benefits

The benefits per ton basalt application depend on future carbon prices:

$$R_N(t) = \Sigma_{k \geq t} P(k)\rho^{-k} \cdot \ [r_{CO_2} w_r \left(1 - w_r\right)^{(k-t)} + CDR_{bio}(k)]$$

Where $CDR_{bio}(k)$ is the marginal biotic capture at time k, P(k) is the price of carbon at time k, ρ is the discount factor, $r_{CO2}$ is the geochemical removal rate (the mass of $CO_2$ removed per ton of rock applied) and $w_r$ is the weathering rate. The quantity $\left(1 - w_r\right)^{(k-t)}$ is the unweathered proportion at time $k$ of the initial basalt applied at time $t$. The revenue of basalt application increases with future carbon prices. On the other hand, the cost of basalt application also increases with the carbon price, which affects the energy cost of basalt application. Therefore, a path of increasing carbon prices is more opportune to enhanced weathering deployment than stable or declining prices. This explains how the marginal revenue can exceed the product of the removal rate by the current carbon price, as well as the decrease of the marginal revenue of basalt application in 2100 (**Supplementary Figure 18**).

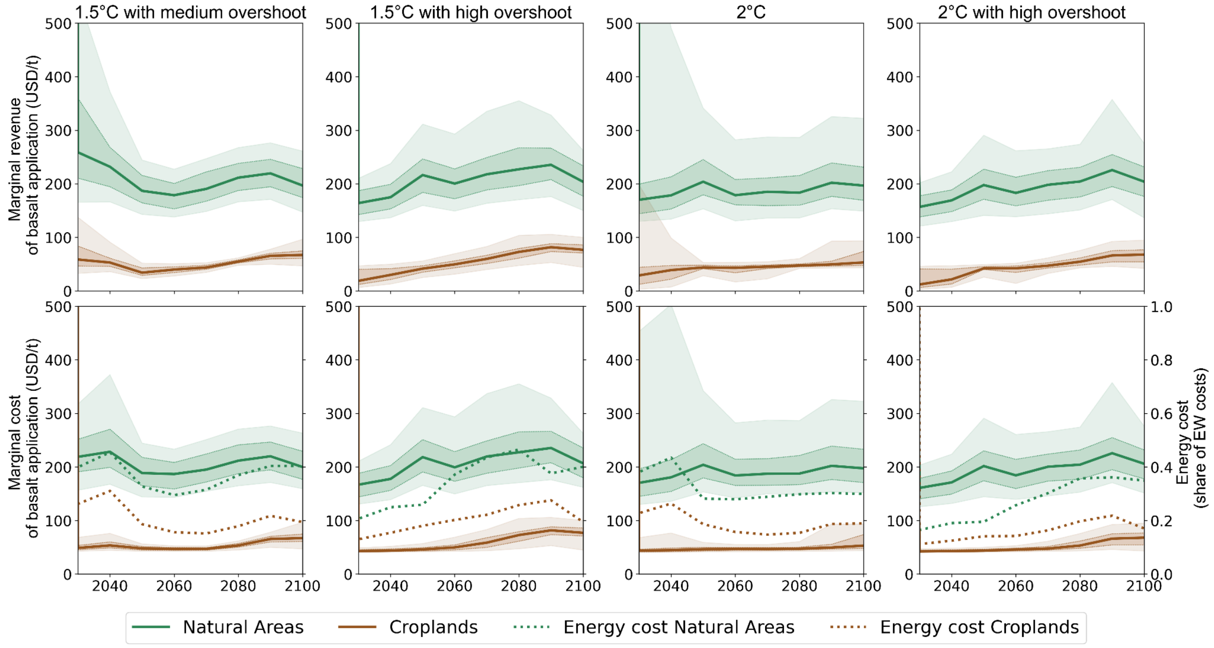


**Supplementary Figure 18 | Marginal revenues and costs of basalt application** in the different climate policy scenarios. Line: median. Shaded areas: 5-95% (light) and 25-75% ranges (dark). The marginal revenue of basalt application is defined as the nominal benefit associated with the CDR resulting from the application of one ton of rock, if no financial or energy expenditure is required. The marginal cost of basalt application is the nominal cost of applying one ton of rock

without any resulting CDR. The energy cost is the nominal price of the energy that would be used to apply one ton of basalt. **The dotted lines** represent the ratio of energy cost over total basalt application cost (**right y-axis**).

### 1.3.3 Hotelling rule and beyond

Due to its non linear behaviour and intrication with the carbon cycle, the term $CDR_{bio}(k)$ is hardly analytically tractable. Let us consider a case where there is no biotic effect ($CDR_{bio} = 0$) and the carbon price increases with the discount rate: $P(t) = \rho^t P_0$ (Hotelling rule), and we thus obtain the very simple expression: $R_N(t) = \rho^t P_0 \Sigma_{k\geq 0} r_{CO_2} w_r (1 - w_r)^k = P(t) r_{CO_2}$, that is to say, the benefit is the product of the $CO_2$ removed per ton of rock by the price of $CO_2$. Since the first ton of rocks applied on croplands costs between \$43 and \$58, and the removal rate is 0.24 to 0.37, the break-even $CO_2$ price ranges from \$116 to \$242 per ton $CO_2$. This is in the range of existing assessments of $CO_2$ removal costs: 80-180\$/t$CO_2$ in ref [33], 200\$/t$CO_2$ in ref [2].

The same analysis where basalt is applied on forests would yield break-even $CO_2$ prices between \$400 and \$1450 per ton $CO_2$ if the biotic effect is not included. Break-even $CO_2$ prices when the biotic effect is included are hard to derive analytically, as the CDR associated with basalt application depends on how much basalt is applied and on previous basalt application. However, one can use the mean removal rate in forests, which is obtained by dividing the total CDR due to enhanced weathering in forests by the cumulative mass of basalt applied over forests over the century. Since the removal rate decreases with increasing basalt application, the scenario where the lowest quantity of basalt is applied displays the highest removal rate. The mean removal rate ranges from 2.2 $t_{CO2}/t_{rock}$ in the 1.5°C with high OS case to 7.2 $t_{CO2}/t_{rock}$ in the 2°C case (**Supplementary Figure 19**). This corresponds to average break-even $CO_2$ prices between \$20 and \$166 per t$CO_2$ depending on the scenario.

The break-even $CO_2$ price is higher if the carbon prices grows slower than the Hotelling rule, as it decreases the benefits of the future removals from current basalt application, and vice-versa. In high OS cases, the $CO_2$ prices increase faster than the discount rate until 2090, and decline afterwards. In medium or no-OS cases, the $CO_2$ prices stabilise when temperature stabilises.

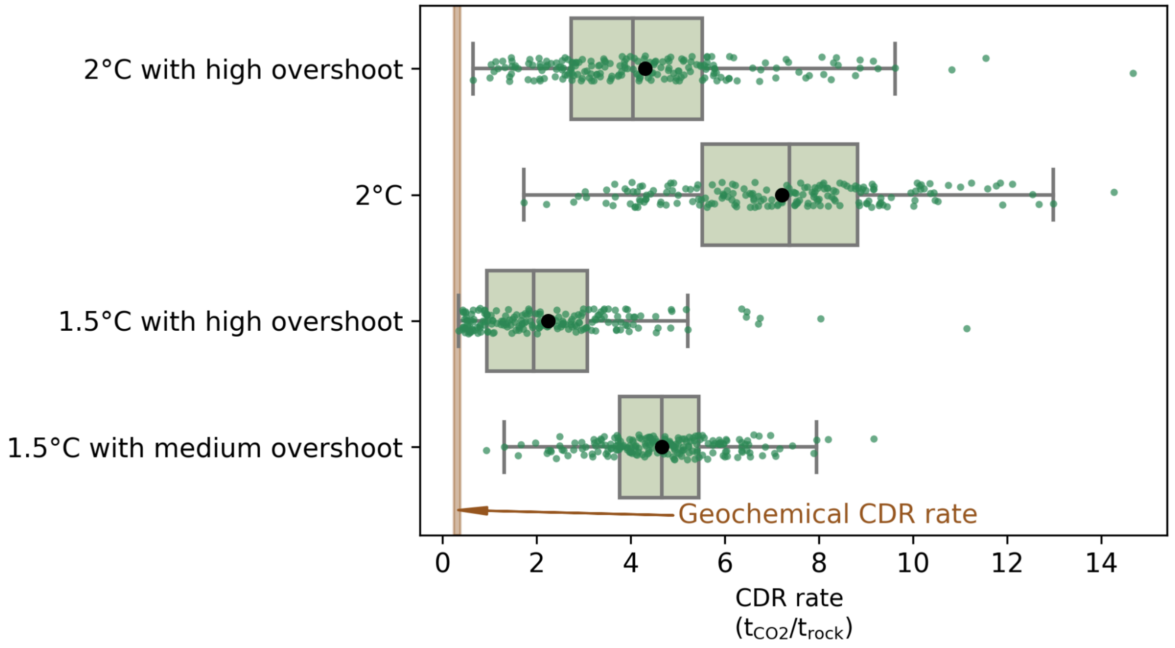


**Supplementary Figure 19 | Mean CDR rate over forests** in the different climate policy scenarios.The boxes represent the 25-75% range, while the vertical bars and black dots inside the boxes represent the median and mean, respectively. The error bars indicate the 5-95% range. Each green dot represents a simulation of the parameters sample. The brown shaded area represents the range of geochemical CDR rate: 0.24-0.37 $t_{CO2}/t_{rock}$.

# 2. Sensitivity Analysis

## 2.1 Parameters distributions

The following table lists the uncertain parameters that are varied in the sensitivity analysis. As many of these parameters have the same kind of influence on the output (e.g. the costs of competing conventional mitigation technologies such as batteries, nuclear power plants & renewables have the same influence), we vary them simultaneously in order to reduce the number of required simulations while covering the full uncertainty space. Parameters are therefore gathered into 10 groups, and are varied together although not in the same direction. For instance, to increase the efficiency of mitigation technology, the costs of low-carbon technologies are decreased while their load factors and maximum penetration levels are increased. The sign of the variation of each parameter relative to the variation of the group is indicated in the table by a (+) or a (-). We have isolated the parameters directly related to enhanced weathering to track their effects independently.

| Parameter | Range | distribution | Justification | Group & variation |
|---|---|---|---|---|
| Energy demand | multiplied by 0.75-1.25 | Uniform | Source: SSP database. The standard deviation of the final energy use in 2100 (across SSP2 scenarios) is assumed to be 25% of its mean value. | Energy demand (+) |

| | | | | |
|---|---|---|---|---|
| Electric share of PHEV use | 0.2-0.69 | Uniform | 0.69 : NEDC, 20% real-world | Efficiency of mitigation technologies (+) |
| Maximum diffusion of technologies (e.g.: maximum share of cogeneration in urban heating systems). | constraints multiplied by 0.9-1.1 | Uniform | | Efficiency of mitigation technologies (+) |
| H2 vehicle costs | Incremental cost (compared to internal combustion engine vehicle): 3,000$/car, 25,020$ for bus and trucks, multiplied by 0.3-2.7 | Uniform | Multiplier range from Cox, et al 2021[34]. | Efficiency of mitigation technologies (-) |
| EV costs (PHEV & BEV) | Incremental cost (compared to internal combustion engine vehicle): 7,200$/car, 62,200$ for bus and trucks, multiplied by 0.9-1.9 | Uniform | Multiplier range from Cox, et al.[34] | Efficiency of mitigation technologies (-) |
| CCS costs | The extra cost of adding CCS to an energy conversion plant (1,340$/kW for gas to electricity, 1,920$/kW for coal to electricity and biomass to electricity, 1,050$/kW for biomass to H2, 1,580$ for biomass to MeOH) is multiplied by 0.8-1.2. | Uniform | The technology readiness level (TRL) is quite low: 6 to 7 (biomass), and 8 to 9 (coal & gas). | Efficiency of CCS (-) |
| Variable renewable energy costs | 1,100$/kW (onshore wind), 895$/kW (solar), multiplied by 0.9-1.1 | Uniform | TRL[5] is high : 9-10 | Efficiency of mitigation technologies (-) |
| Nuclear costs | 5,240$/kW, multiplied by 0.95-1.05 | Uniform | TRL is high : 10 to 11 | Efficiency of mitigation technologies (-) |
| Biomass to H2 or Methanol costs | 6,000$/kW and 3000$/kW for MeOH and H2 (without CCS), | Uniform | Low TRL (5 to 6) | Efficiency of mitigation technologies (-) |

[5] Source for all TRLs : IEA (2022), ETP Clean Energy Technology Guide, IEA, Paris https://www.iea.org/data-and-statistics/data-tools/etp-clean-energy-technology-guide

| | | | | |
|---|---|---|---|---|
| | respectively, multiplied by 0.7-1.3 | | | |
| Electrolysers costs | 1,500$/kW (400$/kW in 2050) multiplied by 0.9-1.1 | Uniform | TRL is quite high (9) | Efficiency of mitigation technologies (-) |
| Storage costs | 2,500$/kW multiplied by 0.85-1.15 | Uniform | TRL 8-9 | Efficiency of mitigation technologies (-) |
| capacity factor of wind and solar | 0.36 for wind, 0.16 solar. multiplied by 0.8-1.00 | Uniform | Coupling of power system model with MESSAGE: slight overestimation of VRE capacity factor in IAMs (Brinkerink, et al. 2022) | Efficiency of mitigation technologies (+) |
| carbon storage cost | 37 $/tC for fossil carbon, 73$/tC for bio carbon, multiplied by 0.85-1.15 | Uniform | Base: GET7.0 parameters, range due to medium TRL (7-8). | Efficiency of CCS (-) |
| Bioenergy crops potential | 160-260 EJ/year, that added to 50EJ of other bioenergy sources (residuals, waste) amount to the range 210-310 EJ/year presented in the text. | Uniform | Global Energy Assessment[35], range from Li, et al (2021)[36] | Bioenergy potential (+) |
| Max. capital growth | 10-20% | Uniform | Base 15% per year (GET7.0 parameter). | Efficiency of mitigation technologies (+) |
| max CCS per year | 10-20 GtCO2/year | Uniform | Range of maximal annual CCS from ENGAGE IAMs in the EN_NPI700 and EN_NPI1000 scenarios (AIM/CGE, MESSAGEix-GLOBIOM, REMIND-MAGPIE): the mean of IAMs maximum annual CCS is 15Gt$CO_2$, the standard deviation of these maximums is 5Gt$CO_2$. | Efficiency of CCS (+) |
| Max growth of fuel supply (transport) | 15% per year, multiplied by 0.5-2 | Uniform | Central value : GET7.0 parameter | Efficiency of mitigation technologies (+) |
| Max intermittent power share | Maximum penetration of wind and solar energy without storage =30%, multiplied by 0.8-1.25 | Uniform | The power system representation is not very detailed in GET7.0 : we assume 30% of VRE penetration without storage or curtailment, and we test | Efficiency of mitigation technologies (+) |

| | | | | |
|---|---|---|---|---|
| | | | the sensitivity of our results to this parameter. | |
| Mean basalt transport distance | 350-550 km | Uniform | see *supra 1.1* | Energy intensity of basalt application (+) |
| Minimum share of basalt road transport | 70-90% | Uniform | Most application sites are in countries where alternative freight modes are not widely available. | Energy intensity of basalt application (+) |
| Weathering rate | 1-26% per year | Uniform | low range: rinder et al. 2021. 3.55 $10^{-12}$ mol/m2/s (corresponding to pH=5.84 T=25°C), $SSA_{BET}$ =0.74 m2/g => 1% dissolved after 1 year for a grain size of 20μm. high range: ORCHIDEE calibration (based on ref[2] for a grain size of 20μm) | Weathering rate (+) |
| CO2 abiotic capture per ton of rock | 0.24 -0.37 $tCO_2/t_{rock}$ | Uniform | T. Amann personal communication. | Geochemical capture rate (+) |
| Basalt Phosphorus content | 0.036%-0.28% | Uniform | Goll et al 2021 | Basalt Phosphorus content (+) |
| Share of basalt applied on natural areas with aircrafts | 70-90% | Uniform | see *supra* | Energy intensity of basalt application (+) |
| Energy use of aircraft application | 1.8-2.5 EJ/Gt | Uniform | see *supra* | Energy intensity of basalt application (+) |
| Electricity required for rock comminution | 0.07-0.6EJ/Gt | quadratic (max= 0.2 EJ/Gt) | Streffler 2018 : for 20μm, 0.2 EJ/Gt is the central estimate, 0.07 low and 0.61 high: we take a quadratic distribution such that the median is 0.2, the max is 0.6 and the min 0.07. | Grinding energy (+) |
| Equilibrium climate sensitivity | lognormal distribution : sigma=0.22,μ=1.12 | lognormal | Sherwood et al., 2020 Assessment of earth's climate sensitivity using multiple lines of evidence. | Climate sensitivity (+) |

For several parameters, we base the cost uncertainty range on the "Technological Readiness Level" (TRL). The Technological Readiness Level (TRL) describes the maturity of a technology or innovation. It provides a scale to evaluate the progress of a technology from its conception to its deployment in real-world application[37]. Although TRL does not explicitly address the cost uncertainty, it reflects the overall progress of a technology, which can influence the accuracy of future costs projections. An exhaustive assessment of cost uncertainty would consider the additional factors such as economies of scale, learning effects, supply chains, labour costs and regulatory requirements that may affect cost estimates.

Source for all TRLs : IEA (2022), ETP Clean Energy Technology Guide, IEA, Paris https://www.iea.org/data-and-statistics/data-tools/etp-clean-energy-technology-guide

## 2.2 Latin hypercube sampling

In the main text, the values are the means of the model simulations performed over a large sample of parameters values. We sample these parameters using a quasi random sampling method, the 'Latin hypercube sampling' method. The idea is as follows: the set of values accessible for each parameter is divided into N (=620) contiguous segments of equal probability. By taking the weighted mean of each segment, we obtain a set of N values $\{v_1^p, v^p_2, ... v^p_N\}$ for each parameter p. For each simulation, each parameter takes a single new value from this set, in a random order. This ensures that the k-dimensional space of parameters is properly covered.

The equilibrium climate sensitivity (ECS) is sampled over 20 values, in order to limit the computational burden: the climate model ACC2 must be re-calibrated (the process called inverse simulation in the model documentation[38]) each time we change the assumption on ECS because a different ECS value implies different optimal values of other parameters to best explain the historical observations given prior information on all parameters[39]. We also run a set of simulations without considering the uncertainty in climate sensitivity: the mean net emission pathways tend to be slightly lower without ECS sampling than with ECS sampling. It is because the relationship between carbon budget for a given temperature target and ECS is not linear, but rather slightly convex.

## 2.3 Morris sampling

We apply the Morris screening method[40–42] to quantify the influence of each parameter on the outputs. Let $X = \{x_1, ..., x_k\}$ be a vector of parameters which are normalised to [0,1], $Y = f(X)$ the output. The idea is to assess the sensitivity of $f$ to each parameter $x_i$ by sampling its 'elementary effects'$EE_i = f\left(x_1, ...x_i + \Delta ... x_k\right) - f(x_1, ...x_k)$ over a sufficiently large set of values of all the $x_j$. Let us call N the size of this set: the procedure must return a sample $\{EE_i^1, ..., EE_i^N\}$ for all i in [1,...,k]. In our case, we chose N=20. The means $\mu_i$ of the elementary effects, their standard deviation $\sigma_i$ and the mean of their absolute values $\mu_i^*$ give useful information about the influence of these parameters. When the computation of $f$ is time-consuming, the Morris sampling procedure can be used to limit the number of required computations for a given value of N. In the sampling procedure, a trajectory starts with a random vector of parameters. We successively increase the value of each parameter in a random order by Δ to create a set of k+1 vectors. For each parameter, we can calculate an elementary effect by subtracting the values that the function f takes between two successive points where the value of that parameter is increased. We then repeat the procedure N times in order to have N elementary effects per parameter.

More formally, a trajectory T is initiated by choosing an initial point $X_0^T$ in $\left[0, \frac{1}{2N-1}, \frac{2}{2N-1}, ..., \frac{N-1}{2N-1}\right]^k$ (there are N possible initial values per parameter), and then iteratively increasing each parameter $i$ by $\frac{N}{2N-1}$ in a random order $\{\sigma^T(i)\}_{i\in[1,p]}$ where $\sigma^T$ is a permutation, to

obtain T=$\{X_1^T, X_2^T \dots X_{k+1}^T\}$. Computing the output along this trajectory yields the elementary effects for each parameter *i:*

$d_i^T = f(X^T_{\sigma(i)+1})$- $f\left(X^T_{\sigma(i)}\right) = f\left(x_1, \dots x_i + \Delta \dots x_k\right) - f(x_1, \dots x_k)$. We produce *N=20* trajectories.

Initial points are sampled following a Latin Hypercube method, and trajectories are chosen to maximise their dispersion and thus their coverage of the parameters space on which elementary effects are computed, following ref.[41]. However, we do not consider the same dispersion function. In ref.[41], The dispersion between two trajectories is the sum of the Euclidean distance of all their points taken pairwise:

$$d_{m,l}= \sum_{i}^{k+1}\sum_{j}^{k+1}\sqrt{\sum_{z}^{k}[X_i^m(z)-X_j^l(z)]^2}$$

where $X_j^l(z)$ is the value of parameter number z at step j of trajectory l. This dispersion measure is too computationally expensive for its purpose. We want, for each parameter *i,* to have a representative set $\{EE_i^1,\dots,EE_i^N\}$ of its elementary effects. Therefore, it is necessary that for each *i,* the $\{X^t_{\sigma^t(i)}\}_{t\in[1,..,N]}$ are well spaced. We therefore propose the following dispersion measure:

$$d_{m,l}= \sum_{i}^{k+1}\sqrt{\sum_{z}^{k}[X_{\sigma^m(i)}^m(z)-X_{\sigma^l(i)}^l(z)]^2}$$

This dispersion measure has the advantage of being less computationally expensive. We want to maximise:

$$ \sum_{m,l} d_{m,l}$$

In our case, we have around k=30 input parameters, hence computing the distance between two trajectories requires around $k^2$ operations, and computing the dispersion requires $(kN)^2$ operations. If we are to pick the N best trajectories out of the $N^k k!$ existing trajectories, it would require to find the best combination of N trajectories among $N^k k!$, which means a total of $\binom{N^{k}k!}{N} k^2 N^2$ operations (around $10^{768}$ for N=20 and k =30). This is not computationally feasible if we want to have N sufficiently high. We improve the sampling strategy by using a simulated annealing algorithm instead of a brute force approach, which greatly reduces the computational burden.

**Simulated annealing:**

*initialization*

Take N initial points of k parameters with LHS. We sample only over the inferior half-section of each dimension for symmetry reasons. It yields N initial points with coordinates in $[0,\frac{1}{2(N-1)},\dots,\frac{N-1}{2(N-1)}]$, and from each point we will start one trajectory by

generating random permutations $\{\sigma^1,\dots,\sigma^N\}$. Each trajectory is built step by step by adding $$\Delta = \frac{N}{2N-1}$$ to each coordinate in the order $\{\sigma^1(1), \dots \sigma^1(k)\}$. We call $J_p^0=\{t^0_1 \dots t^0_N\}$ this first set of trajectories.

Distance table : compute, for each trajectory $t^0_i$ the distance $d^0_i = \Sigma\ d(t_i^0, t_j^0)$ (we want to maximise $D = \Sigma\ d_i$)

T is the "temperature". λ is the cooling factor.

*While n < Iterations limits:*

*step n*

- T=$\lambda T$
- Pick a random integer $i \in [1,\dots, N]$ . Generate a new random permutation. Let us call $t^{\sim}_i$ the trajectory built from point $i$ and this permutation. Replacing $t^{n-1}_i$ by $t^{\sim}_i$ would increase the dispersion by $\Theta = \Sigma\ d(t_i^{\sim}, t_j^{n-1}) - d^{n-1}_i$.
- If $\Theta \geq 0$
  - Replace $t_j^{n-1}$by $t_i^{\sim}$ and update the distance table.
- If $\Theta$ <0
  - With probability $e^{-\frac{\Theta}{T}}$:
    - Replace $t_j^{n-1}$by $t_i^{\sim}$ and update the distance table.

*End*

Return the best set of trajectories from all those explored.

## 2.4 Discount rate

In addition to the discount rate of the main text (5% per year), we reproduced the results with a discount rate of 2% per year.

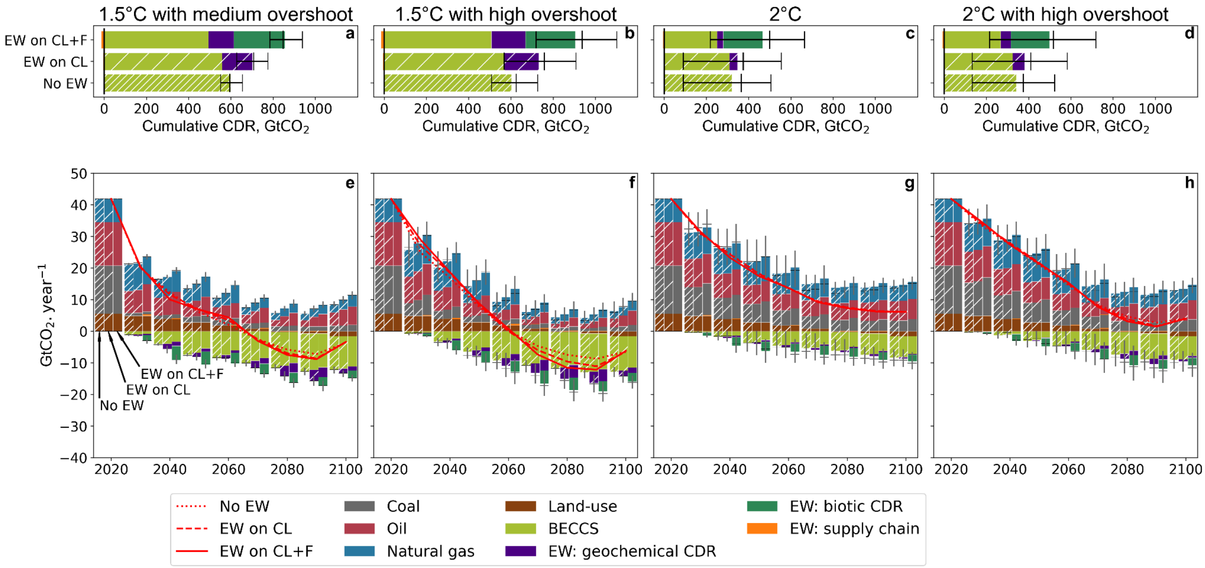


**Supplementary Figure 20| Carbon dioxide emissions across the 21st century for different climate targets, with a discount rate of 2% per year.** The red lines represent the net $CO_2$ emissions from the energy system. Four climate policy targets are compared (one per column): **1.5°C with medium overshoot**: The temperature change is limited to 1.5°C after 2100, with a possible overshoot of up to 0.2°C before 2100. **1.5°C with high overshoot:** the temperature change is limited to 1.5°C after 2100. **2°C with no overshoot**: the temperature change is limited to 2°C. **2°C with high overshoot:** the temperature change is limited to 2°C after 2100. Three NET portfolios are assessed: **No EW**: BECCS only. **EW on CL:** EW deployed on croplands only, and BECCS. **EW on CL+F:** EW deployed on croplands and forest areas, and BECCS. The bars indicate the mean, the black dashes the median and the 25%-75% range.

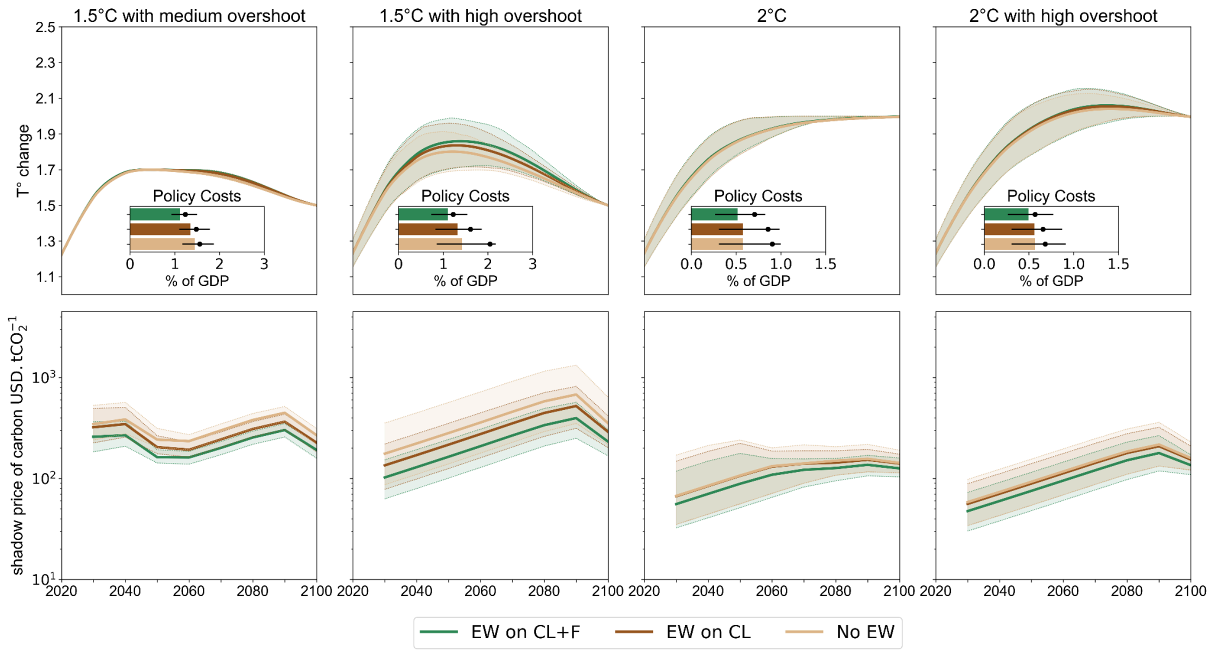


**Supplementary Figure 21 | Discount rate of 2% per year. Top:** Mean surface temperature change compared to preindustrial period. **Bottom: Shadow price of carbon across the 21st century for different climate targets.** The shaded area represents the 25%-75% range. The scale changes between the panels. **Policy costs** are the net present values of future energy production costs and consumption losses as a percentage of GDP, compared to the no-policy scenario.

# 3. GET-ACC2 model

## 3.1 Overview of the model

The GET-ACC2 model is an integrated climate-energy-economic single-region model with global coverage. It is used for long-term energy system modelling and technology assessment. It describes how the energy system evolves and minimises its costs across the 21st century to meet the energy demand and respect specified constraints, such as a carbon budget or a climate target. GET-ACC2 is composed of two models: the energy system model GET and the reduced complexity climate model ACC2.

The Aggregated Carbon Cycle, Atmospheric Chemistry, and Climate (ACC2) model describes the physical part of the system. K. Tanaka developed the model in 2007. It computes the evolution of physical parameters, such as greenhouse gas concentrations in the atmosphere, the additional radiative forcing that it induces, and the surface mean temperature resulting from a pathway of anthropogenic emissions. The ACC2 model is clearly and comprehensively described in the documentation by Katsumasa Tanaka and Elmar Kriegler[38,43].

The global energy transition (GET) model is a simple linear energy system model that was initially developed at Chalmers University by Christian A. Azar and Kristian Lindgren[44,45]. The current version, GET7.1, is derived from the one used by D.J.A. Johansson and C. Azar in Azar *et al* (2013) and [46]). More information on GET7.0 can be found in ref[47].

The GET-ACC2 model computes cost-optimal scenarios to balance energy demand with energy supply. It takes as input a set of assumptions about future demand in several subsectors, technology costs and efficiency, and returns a combination of energetic and economic features of the cost-optimal energy mix, such as primary energy needs, marginal and total costs of production and

distribution, etc. The model has 4 kinds of input data: techno-economical parameters, that characterise the costs and efficiencies of the technologies included in the model, climate model parameters, assumptions on future energy demand, and climate policy constraints.

The energy demand comprises four sectors for stationary end-use: industrial process heat, electricity, feed-stock for chemical industry, residential and commercial heating, and eight transport demand subsectors: road (public & private transport), air and rail for passenger transport, and marine, air, rail and road for freight. These energy demand pathways are derived from ENGAGE SSP2 baseline scenarios[48]. The GET model computes, at each 10-year timestep, new productive capacity, new vehicles, distribution infrastructure etc. that have to be deployed to meet a given demand, as well as the fluxes of primary and secondary energy, the emission of greenhouse gases that stem from them, and costs incurred by the system. The reference year for dollar value is 2010.

### 3.1.1 Optimisation procedure

The model is solved by a non-linear optimization solver, CONOPT4. There are two options to solve the model: the demand can be either fixed or price-responsive. The solver minimises the objective function: the net present value of future costs (investment costs, O&M costs, fuel costs, carbon tax, etc.), when the demand is fixed, or the net present value of annual surplus, when demand is price-responsive. The discount rate is assumed to be at 5% by default. The solver ensures that the climate constraints are respected - for instance, keeping global warming from pre-industrial level below 1.5°C in 2100. In this study, we use the price-responsive mode.

To allow the demand to decrease when prices grow, price-elasticities are -0.4 for electricity demand[49], domestic and commercial heating, -0.2 for industry (process heat and feed-stock), and -0.3 for transport demand [50,51].

The model is solved twice:

- A first run with fixed demand and no climate policy $Q_b^s$ (*b* for "baseline", *s* for "sector") yields baseline end-use prices $P_b^s$ at each 10-year timestep, for each sector (the marginal costs of energy supply). Here, the net present value $O_{fixed}$ of future energy system costs C(t) is minimised:

$$O_{fixed} = \sum_t C(t)\rho^{-(t-t_0)} \text{ where } \rho = 1.05$$

These prices are considered the reference prices for the demand pathway, and we deduce the reactive demand curve $Q_r^s$ (*r* for "reactive") from the ratio between reference prices and real prices.

$$\frac{dlog(Q)}{dlog(P)} = \eta \text{ hence } Q_r^s = Q_b^s\left(\frac{P_r^s}{P_b^s}\right)^{\eta} \text{ where } \eta \text{ is the price-elasticity of demand.}$$

- A second run (with elastic demand) computes the consumer surplus of each sector at each 10-year timestep, that is to say the area below the demand curve. The annual social surplus S(t) is the consumer surplus minus the production costs, i.e the area below the supply curve:

$$S(t) = \sum_{sectors} \left[ \left( \frac{Q_r^{\ s}(t)}{Q_b^{\ s}(t)} \right)^{\frac{1}{\eta_s}} \frac{P_b^{\ s}(t)}{\frac{1}{\eta_s} + 1} Q_r^{\ s}(t) \right] - C(t)$$

The solver maximises the discounted sum $O_{reactive}$ of the annual social surplus between 2020 and 2150.

$$O_{reactive} = \sum_{t} S(t) \rho^{-(t - t_0)} \text{ where } \rho = 1.05$$

As a consequence, strengthening climate policies increases energy costs and the energy demand is reduced compared to a No-Policy baseline (Supplementary Figure 22).

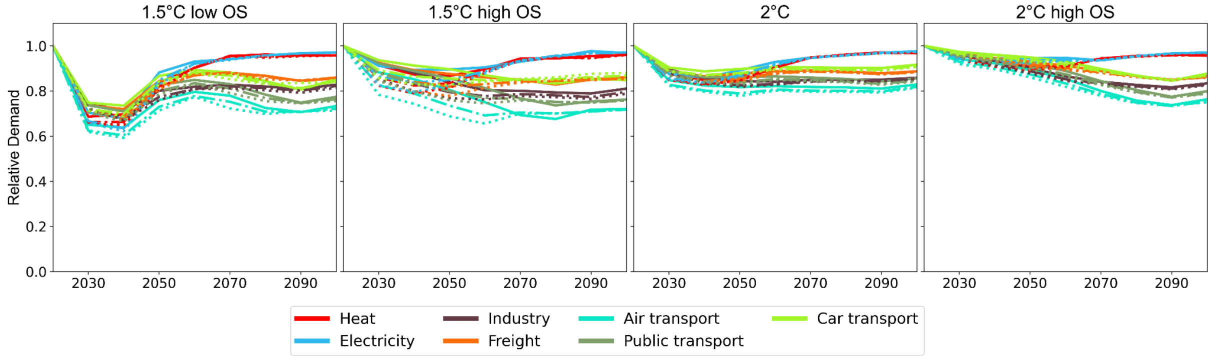


**Supplementary Figure 22** Top: Relative energy demand in mitigation scenarios compared to the baseline. Bottom: Relative biomass price compared to the scenarios without enhanced weathering (EW). Solid line: EW deployed on croplands (CL) and forests (F), and bioenergy with carbon capture and storage (BECCS). Dash-dotted line: EW on CL only, and BECCS. Dotted line: BECCS only.

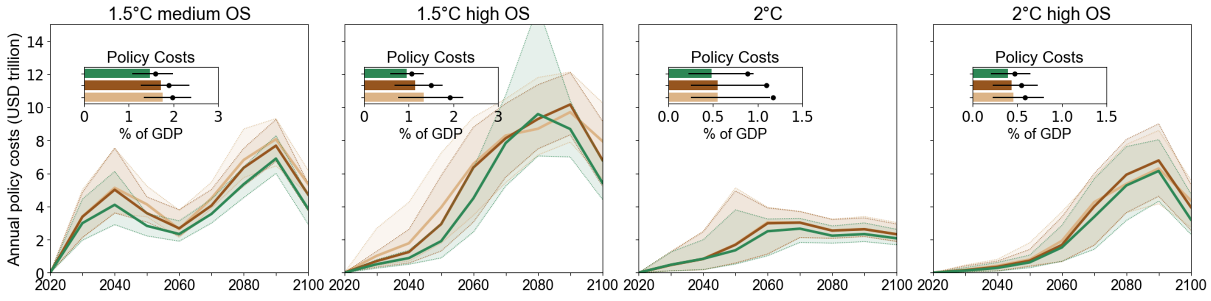


**Supplementary Figure 23 | Annual median mitigation costs in GET-ACC2** (discount rate = 5%). The dotted lines show the 25-75% range.

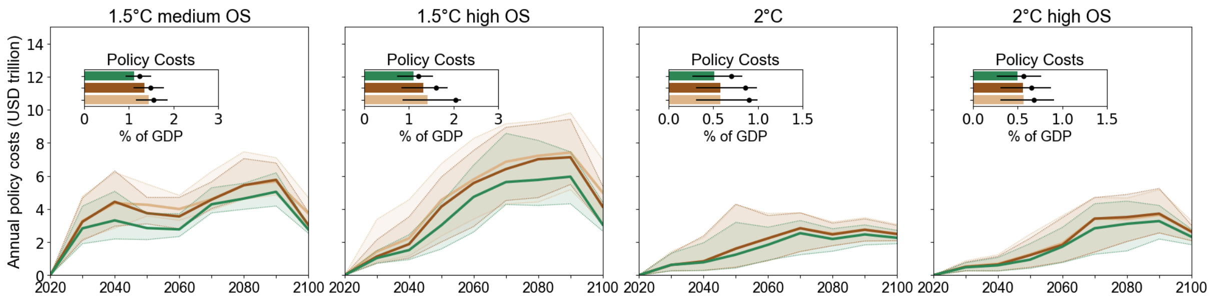


**Supplementary Figure 24| Annual median mitigation costs in GET-ACC2** with discount rate =2% per year. The dotted lines show the 25-75% range.

### 3.1.2 Model Validation

The climate model ACC2[38,39,43,52–56] is calibrated on historical data[38], and its temperature response to greenhouse gas emissions has been validated against similar reduced-complexity climate models [57]. The carbon sequestration following an increase in net primary production in ACC2 has been validated against CMIP6 models in the context of an increase in net primary production due to CO2 fertilisation[55], but not phosphorus fertilisation which is a process (to date) absent from all CMIP6 models.

The land-surface model ORCHIDEE-CNP was well evaluated from site to global scale including nutrient leaching from terrestrial soils and the effects of elevated CO2 on primary productivity and land carbon storage [19,58–60]. Further, the response of aboveground productivity to mineral P fertiliser addition was compared with observation-based estimates[18]. The emulator that reproduces the NPP response of ORCHIDEE-CNP to basalt addition, relying on a reduced set of assumptions for extrapolating the behaviour of the complex ORCHIDEE-CNP model, is necessarily a simplification of the original model but we checked that it reproduces faithfully the emerging response of the complex model.

The energy system model GET was developed for providing least-cost scenarios of energy transition [44–47,61,62]. The initial state of the energy system is calibrated on IEA data regarding energy flows and production capacities, and technology costs are taken from other energy system models. This kind of forward-looking model is hard to validate[63] because it does not intend to make predictions about the future, but rather to provide quantitatively self-consistent energy scenarios, in an idealised world where a central planner with perfect foresight coordinates the energy transition to achieve climate targets at the lowest cost.

## 3.2 Major updates on GET

The model GET7.0 is described in ref[47]. The model GET7.1, used in this study, was updated on several aspects: supply curves for fossil fuels and bioenergy, marginal abatement costs curves for non-$CO_2$ gases, the investment costs were updated to account for the rapid decline in wind turbine and solar PV costs as well as batteries.

### 3.2.1 Fossil fuel supply curves

The version GET7.0 assumed a constant cost for fossil fuel extraction. The GET7.1 includes fossil fuels supply curves that represent how costs increase as resources are depleted. These supply curves are derived from the TIAM-UCL model[64].

### 3.2.2 Biomass supply curve

The version GET7.0 assumed a constant cost for bioenergy supply. The GET7.1 model does not model land use, but represents the increasing marginal costs of bioenergy supply. We use an idealised supply curve based on the one computed with the IMAGE3.0[65] model (Figure 6 of ref [65]). We assume that the supply curve follows the equation : $P = P_{max}\, Q^4 / Q_{max}^4 + b$, where $b$ is the initial bioenergy price, $P_{max}$ =15$2005/GJ, and $Q_{max}$ is the assumed maximum bioenergy supply potential (between 160 and 260 EJ/year). This simple supply curve generally underestimates the biomass supply cost compared to the original data, but it remains an improvement compared to the initial static cost parameterization.

There are two sources of carbon dioxide emissions in the biomass supply process. First, the machinery used for cultivation, management and harvesting of the bioenergy crops can be powered by fossil fuels. In the GET model, the exogenous energy demand pathways are assumed to take account of the use of energy to produce energy (e.g., for the extraction of materials used to produce renewable energy), thus switching from coal to biomass does not increase the energy demand. The low density of the biomass and the additional transport requirements are accounted for through a financial cost but not an energy one. Second, the extension of cultivated lands to increase the biomass supply could affect the vegetation and soil carbon stocks, possibly increasing land-use change emissions [66,67]. However, the variation in soil carbon stock can be positive or negative depending on the type of land and the type of crops[68]. As a consequence, the governance and regulation of the land-use sector is critical to limit land use change emissions associated with bioenergy production, notably induced direct and indirect land use change[69]. Here, consistently with the perfect information, perfect foresight, optimising paradigm of the model, we assume that bioenergy crop areas are chosen wisely in order to minimise land-use change emissions. This corresponds to an emission factor of 5 $kgCO_2$ per GJ of primary energy (12 $kgCO_2$ per GJ of biofuels converted with 41% efficiency from primary energy[69]). Other land use emissions are exogenous.

### 3.2.3 Methane and Nitrous oxide abatement

New abatement cost curves to reduce methane and nitrous oxide emissions are implemented.There are 4 source-dependent abatement cost curves for methane (for coal, oil, natural gas and non-energy emissions), and one for $N_2O$.

$CH_4$ emissions from the energy sector are the product of the quantities of natural gas, oil and coal by the corresponding emission factors (0.275, 0.22, and 0.284 $MtCH_4$ per EJ of gas, oil and coal respectively). These three emission factors can be reduced at the expense of additional costs that are derived from marginal abatement costs curves[70]. Additionally, non-energy methane emissions follow an exogenous baseline based on SSP2. Non-energy methane emissions can also be reduced at the expense of an additional abatement cost[70].

Bioenergy supply is associated with $N_2O$ emissions (0.01 MtN per EJ). Bioenergy and non-energy (exogenous) $N_2O$ emissions can also be reduced similarly to $CH_4$ mitigation.

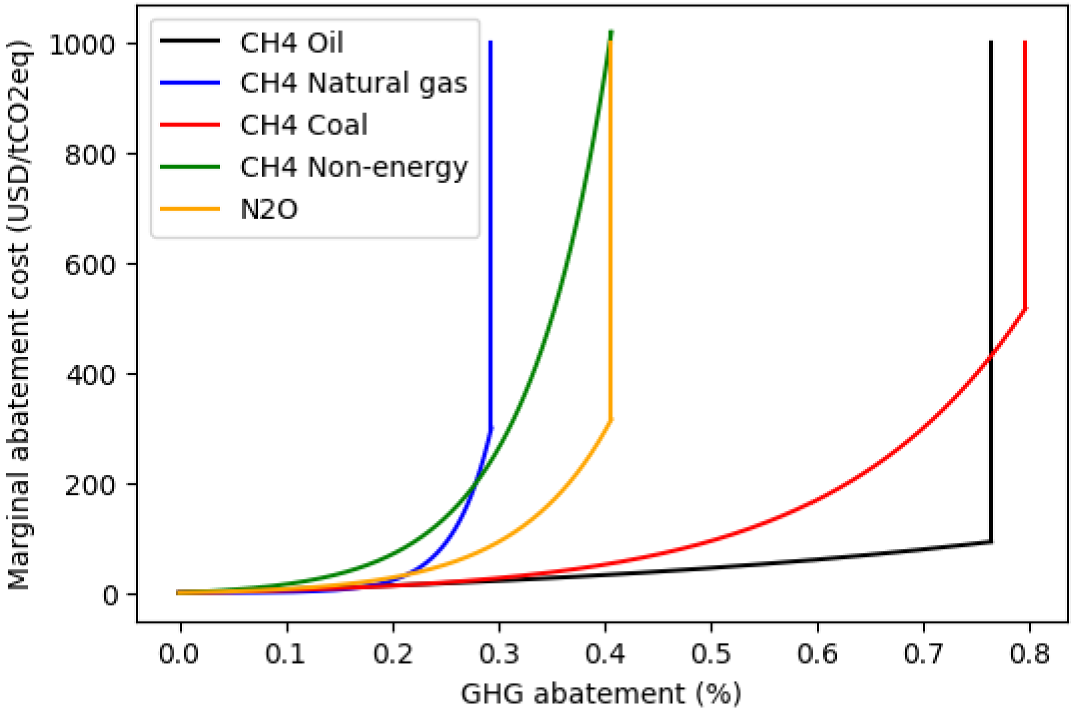


**Supplementary Figure 25 | Marginal abatement cost curves for $CH_4$, and $N_2O$**

### 3.2.4 Parameters updates

As solar panels, wind turbines and batteries costs have declined, the capital costs in GET7.1 are different from those in GET7.0. Electricity generation costs are based on the data from EIA, NREL, IRENA[71–73], while H2 and MeOH generation costs are based on the REMIND model[74]. The electric vehicles and plug-in hybrid electric vehicles are also cheaper, as we assume a battery cost of 100$/kWh.

The total CO2 storage reserves were extended to 3000 $GtCO_2$. A maximum annual injection rate of 0.5% of storage reserves was added, following ref.[32] This leads to a maximum annual $CO_2$ storage of 15$GtCO_2$ per year, consistent with annual sequestration rates in the ENGAGE database[75] Furthermore, the extension of annual CCS flows is limited to 150 MtC/year, i.e. 5.5 $GtCO_2$ per decade.

### 3.2.4 Renewable energy mix

The onshore wind power potential is 80EJ/year[76]. There is no detailed description of wind and solar production in GET, nor of issues related to VRE penetration in power production such as curtailment. The maximum share of variable renewable energy that does not have to be stored is limited to 30%, and the rest can be freely integrated as long as batteries (or cheaper storage with limited potential, such as dams) are purchased. We imposed that the shares of variable renewable energy provided by wind or solar energy cannot exceed given thresholds. Otherwise, since wind power is cheaper than solar, it completely replaces it.

## 3.3 Comparison of mitigation scenarios and baseline

When no climate target nor carbon tax constrains the model, energy supply relies heavily on coal, although the shares of wind, solar and nuclear power also increase (Supplementary Figure 26). The $CO_2$ emissions rise steadily across the 21$^{st}$ century and peak around 2100 (Supplementary Figure 27).

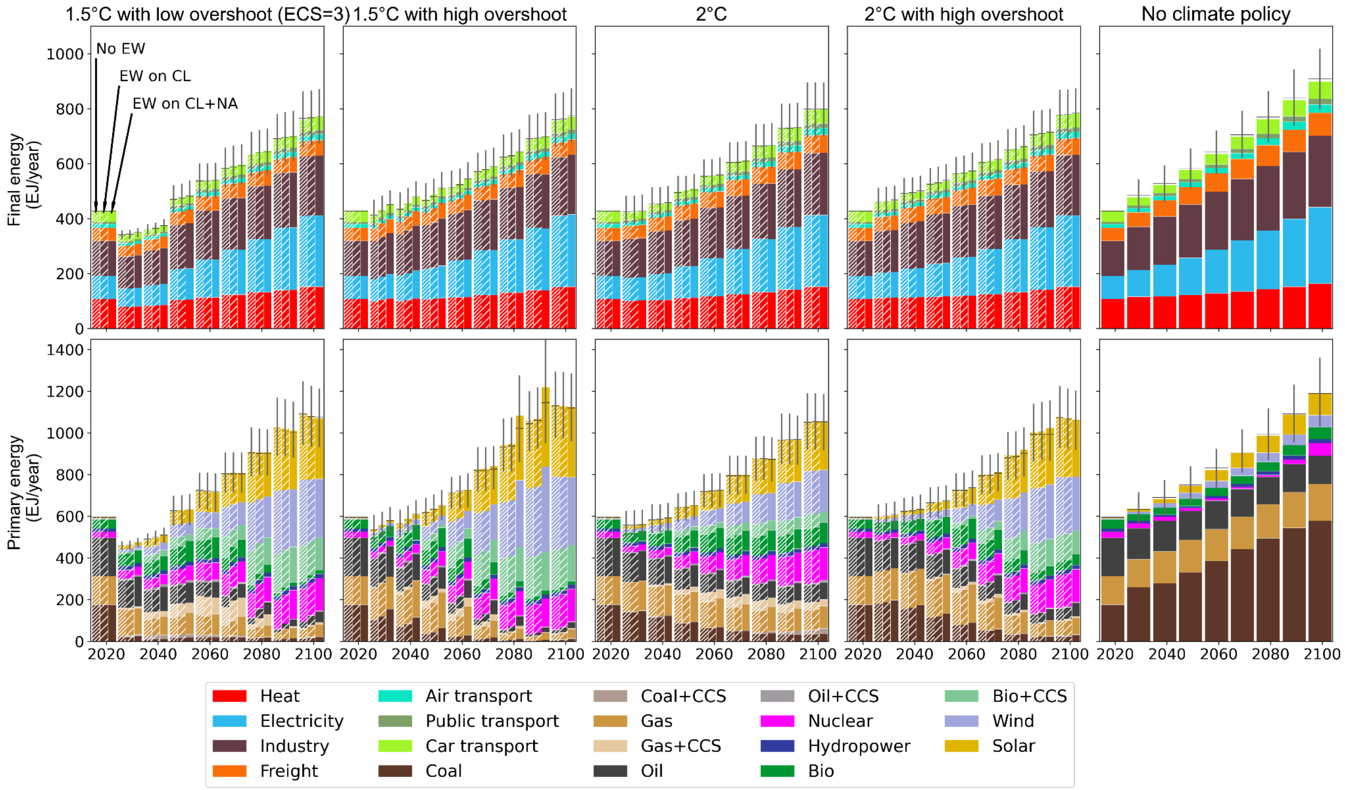


**Figure 26| Energy flows in GET-ACC2**. The hatched bars follow the same pattern as in the main text, except for the right panel where no EW is applied. The error bars represent the 25-75% range.

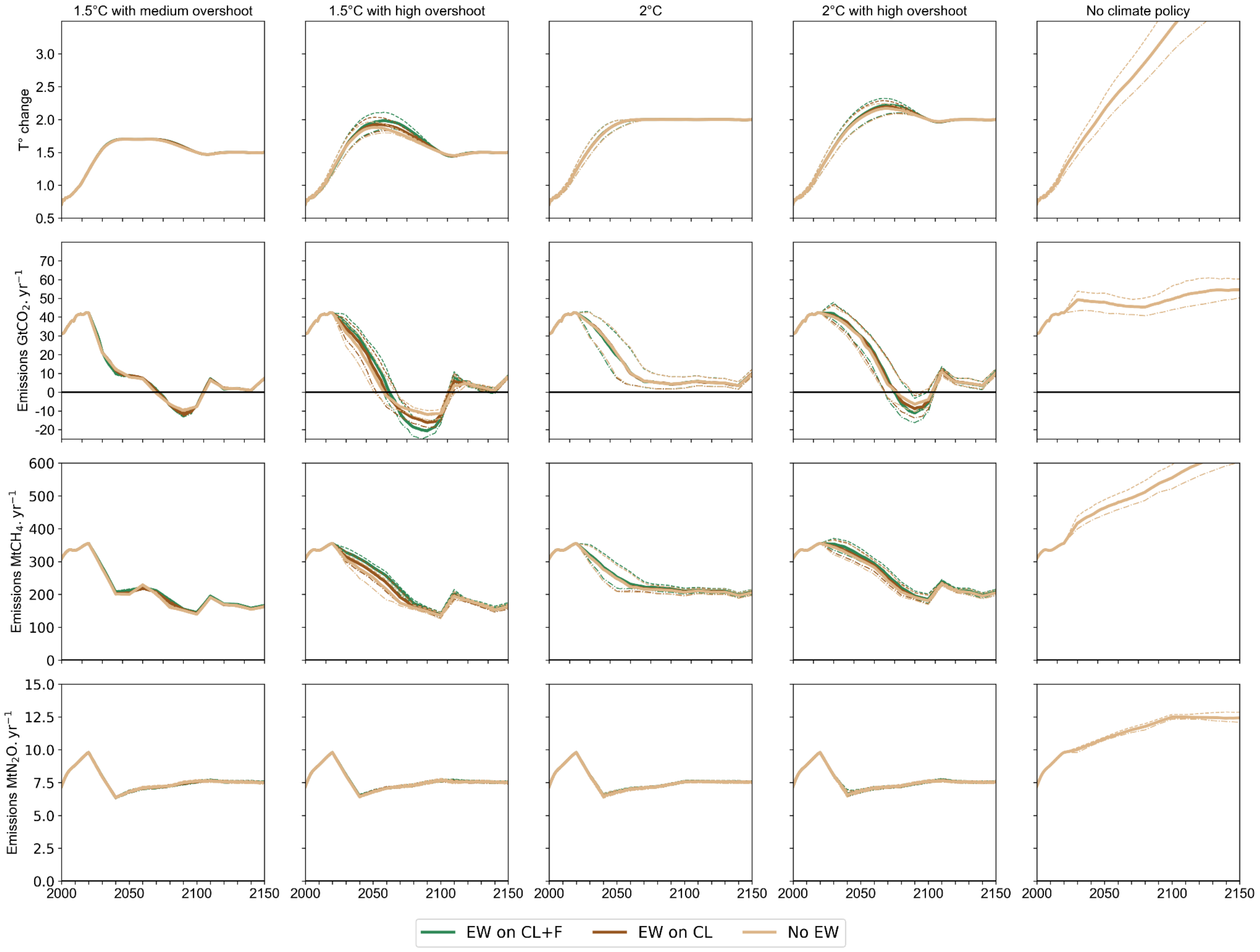


**Supplementary Figure 27 | GHG Emissions and Temperature change in GET-ACC2**. The dotted lines show the 25-75% range. When the temperature returns to its target after overshoot, the climate inertia leaves a little space for emissions to bounce back, while keeping the temperature below target. As the calculation is made up to 2150, emissions increase slightly in 2150 because they do not have enough time to affect the climate system: it is a side-effect due to the assumed time horizon.

## 3.4 Inverse parameterisation of the climate model ACC2

The spread of uncertainty relating to the climate system is assessed by sampling the equilibrium climate sensitivity[77] (ECS). In ACC2, several parameters are jointly calibrated on historical values using the inverse mode[38]. Here the value of the ECS is exogenously set, and other parameters are calibrated consistently to this ECS value. The most influential parameters are Q10, by which the rate of terrestrial heterotrophic respiration increases with a temperature increase of 10°C, the factor β, which logarithmically scales the $CO_2$ fertilisation effect on net primary production with the fractional change of atmospheric $CO_2$ concentration, and the aerosol forcing. The **Supplementary Figure 28** shows how these parameters vary with the ECS.

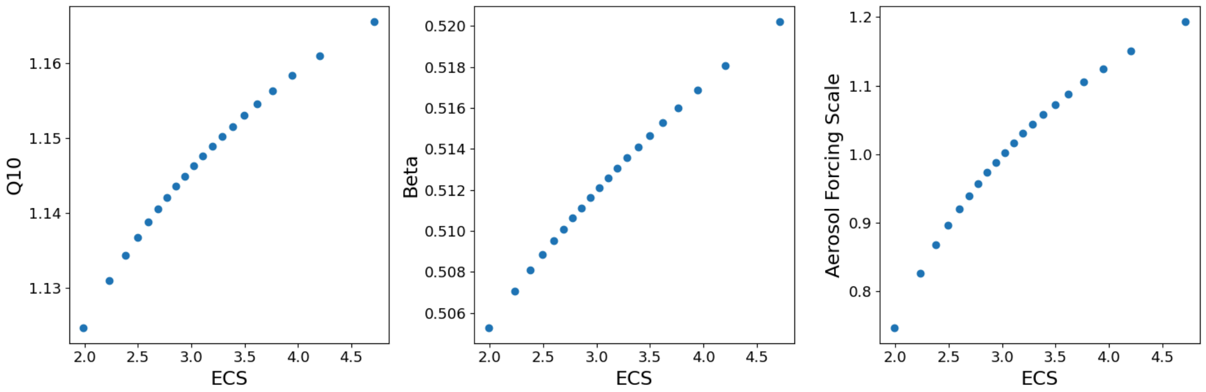


**Supplementary Figure 28 | Correlations between climate parameters Q10, Beta and the Aerosol forcing**, as estimated from ACC2 inverse mode.

*References*


1. Rinder, T. & von Hagke, C. The influence of particle size on the potential of enhanced basalt weathering for carbon dioxide removal - Insights from a regional assessment. *Journal of Cleaner Production* **315**, 128178 (2021).
2. Strefler, J., Amann, T., Bauer, N., Kriegler, E. & Hartmann, J. Potential and costs of carbon dioxide removal by enhanced weathering of rocks. *Environ. Res. Lett.* **13**, 034010 (2018).
3. Vanderkloot, E. & Ryan, P. Quantifying the effect of grain size on weathering of basaltic powders: Implications for negative emission technologies via soil carbon sequestration. *Applied Geochemistry* **155**, 105728 (2023).
4. Ryan, P. C. *et al.* The potential for carbon dioxide removal by enhanced rock weathering in the tropics: An evaluation of Costa Rica. *Science of The Total Environment* **927**, 172053 (2024).
5. Amann, T. *et al.* Enhanced Weathering and related element fluxes – a cropland mesocosm approach. *Biogeosciences* **17**, 103–119 (2020).
6. Berge, H. F. M. ten *et al.* Olivine Weathering in Soil, and Its Effects on Growth and Nutrient Uptake in Ryegrass (Lolium perenne L.): A Pot Experiment. *PLOS ONE* **7**, e42098 (2012).
7. Dietzen, C., Harrison, R. & Michelsen-Correa, S. Effectiveness of enhanced mineral weathering as a carbon sequestration tool and alternative to agricultural lime: An incubation experiment. *International Journal of Greenhouse Gas Control* **74**, 251–258 (2018).
8. Larkin, C. S. *et al.* Quantification of CO2 removal in a large-scale enhanced weathering field trial on an oil palm plantation in Sabah, Malaysia. *Front. Clim.* **4**, 959229 (2022).
9. Haque, F., Santos, R. M. & Chiang, Y. W. CO2 sequestration by wollastonite-amended agricultural soils – An Ontario field study. *International Journal of Greenhouse Gas Control* **97**, 103017 (2020).
10. Reershemius, T. *et al.* Initial Validation of a Soil-Based Mass-Balance Approach for Empirical Monitoring of Enhanced Rock Weathering Rates. *Environ. Sci. Technol.* **57**, 19497–19507 (2023).
11. Kelland, M. E. *et al.* Increased yield and $CO_2$ sequestration potential with the $C_4$ cereal *Sorghum bicolor* cultivated in basaltic rock dust‐amended agricultural soil. *Glob Change Biol* **26**, 3658–3676 (2020).

12. Beerling, D. J. *et al.* Enhanced weathering in the US Corn Belt delivers carbon removal with agronomic benefits. *Proc. Natl. Acad. Sci. U.S.A.* **121**, e2319436121 (2024).
13. Buckingham, F. L., Henderson, G. M., Holdship, P. & Renforth, P. Soil core study indicates limited CO2 removal by enhanced weathering in dry croplands in the UK. *Applied Geochemistry* **147**, 105482 (2022).
14. Lewis, A. L. *et al.* Effects of mineralogy, chemistry and physical properties of basalts on carbon capture potential and plant-nutrient element release via enhanced weathering. *Applied Geochemistry* **132**, 105023 (2021).
15. Wang, F. *et al.* Wollastonite powder application increases rice yield and CO2 sequestration in a paddy field in Northeast China. *Plant Soil* (2024) doi:10.1007/s11104-024-06570-5.
16. West, L. J., Banwart, S. A., Martin, M. V., Kantzas, E. & Beerling, D. J. Making mistakes in estimating the CO2 sequestration potential of UK croplands with enhanced weathering. *Applied Geochemistry* **151**, 105591 (2023).
17. Buckingham, F. L., Henderson, G. M. & Renforth, P. Response to Comment from West et al. on, "Soil core study indicates limited CO2 removal by enhanced weathering in dry croplands in the UK". *Applied Geochemistry* **152**, 105622 (2023).
18. Goll, D. S. *et al.* Potential CO2 removal from enhanced weathering by ecosystem responses to powdered rock. *Nat. Geosci.* **14**, 545–549 (2021).
19. Goll, D. S. *et al.* A representation of the phosphorus cycle for ORCHIDEE (revision 4520). *Geoscientific Model Development* **10**, 3745–3770 (2017).
20. Virtanen, P. *et al.* SciPy 1.0: Fundamental Algorithms for Scientific Computing in Python. *Nature Methods* **17**, 261–272 (2020).
21. Atima, D. & Suthirat, K. Estimated Greenhouse Gases Emissions from Mobile and Stationary Sources in the Limestone and Basalt Rock Mining in Thailand. *American Journal of Environmental Sciences* **12**, (2016).
22. Renforth, P. The potential of enhanced weathering in the UK. *International Journal of Greenhouse Gas Control* **10**, 229–243 (2012).
23. Thrikawala, S., Weersink, A., Fox, G. & Kachanoski, G. Economic Feasibility of Variable-Rate

Technology for Nitrogen on Corn. *American Journal of Agricultural Economics* **81**, 914–927 (1999).

24. Ibisch, P. L. *et al.* A global map of roadless areas and their conservation status. *Science* **354**, 1423–1427 (2016).
25. Hartmann, J. & Moosdorf, N. The new global lithological map database GLiM: A representation of rock properties at the Earth surface. *Geochemistry, Geophysics, Geosystems* **13**, (2012).
26. M. C. E. Grafton, I. J. Yule, C. E. Davies, R. B. Stewart, & J. R. Jones. Resolving the Agricultural Crushed Limestone Flow Problem from Fixed-Wing Aircraft. *Transactions of the ASABE* **54**, 769–775 (2011).
27. Bošeľa, M. & Šebeň, V. Analysis of the aerial application of fertilizer and dolomitic limestone. *Journal of Forest Science* **56**, 47–57 (2018).
28. Clair, T. A. & Hindar, A. Liming for the mitigation of acid rain effects in freshwaters: A review of recent results. *Environ. Rev.* **13**, 91–128 (2005).
29. Moraes, T. R., Cornago Junior, V. M., Araújo, V. C. R. de, Esperancini, M. S. T. & Antuniassi, U. R. COST OF AERIAL AND GROUND SPRAYINGS AND TECHNOLOGICAL REPLACEMENT POINT: A CASE STUDY IN THE REGION OF MINEIROS, GO, BRAZIL. *Eng. Agríc.* **41**, 359–367 (2021).
30. Taylor, L. L. *et al.* Enhanced weathering strategies for stabilizing climate and averting ocean acidification. *Nature Clim Change* **6**, 402–406 (2016).
31. Fuhrman, J. *et al.* Diverse carbon dioxide removal approaches could reduce impacts on the energy–water–land system. *Nat. Clim. Chang.* **13**, 341–350 (2023).
32. Strefler, J. *et al.* Carbon dioxide removal technologies are not born equal. *Environ. Res. Lett.* **16**, 074021 (2021).
33. Beerling, D. J. *et al.* Potential for large-scale CO2 removal via enhanced rock weathering with croplands. *Nature* **583**, 242–248 (2020).
34. Cox, B., Bauer, C., Mendoza Beltran, A., van Vuuren, D. P. & Mutel, C. L. Life cycle environmental and cost comparison of current and future passenger cars under different energy scenarios. *Applied Energy* **269**, 115021 (2020).

35. Rogner, H.-H. *et al.* Chapter 7 - Energy Resources and Potentials. in *Global Energy Assessment - Toward a Sustainable Future* 423–512 (Cambridge University Press, Cambridge, UK and New York, NY, USA and the International Institute for Applied Systems Analysis, Laxenburg, Austria, 2012).

36. Li, W. *et al.* Bioenergy Crops for Low Warming Targets Require Half of the Present Agricultural Fertilizer Use. *Environ. Sci. Technol.* **55**, 10654–10661 (2021).

37. Mankins, J. Technology Readiness Level – A White Paper. (1995).

38. Tanaka, K. *et al.* Aggregated Carbon cycle, atmospheric chemistry and climate model (ACC2): description of forward and inverse mode. 14069106 (2007) doi:10.17617/2.994422.

39. Tanaka, K., Raddatz, T., O'Neill, B. C. & Reick, C. H. Insufficient forcing uncertainty underestimates the risk of high climate sensitivity. *Geophys. Res. Lett.* **36**, L16709 (2009).

40. King, D. M. & Perera, B. J. C. Morris method of sensitivity analysis applied to assess the importance of input variables on urban water supply yield – A case study. *Journal of Hydrology* **477**, 17–32 (2013).

41. Campolongo, F., Cariboni, J. & Saltelli, A. An effective screening design for sensitivity analysis of large models. *Environmental Modelling & Software* **22**, 1509–1518 (2007).

42. Morris, M. D. Factorial Sampling Plans for Preliminary Computational Experiments. *Technometrics* **33**, 161–174 (1991).

43. Tanaka, K. & O'Neill, B. C. The Paris Agreement zero-emissions goal is not always consistent with the 1.5 °C and 2 °C temperature targets. *Nature Climate Change* **8**, 319–324 (2018).

44. Azar, C. A., Lindgren, K. & Andersson, B. Hydrogen Or Methanol in the Transportation Sector? (2000).

45. Azar, C. & Lindgren, K. Global energy scenarios meeting stringent CO2 constraints— cost-effective fuel choices in the transportation sector. *Energy Policy* 16 (2003).

46. Johansson, D. J. A., Azar, C., Lehtveer, M. & Peters, G. P. The role of negative carbon emissions in reaching the Paris climate targets: The impact of target formulation in integrated assessment models. *Environ. Res. Lett.* **15**, 124024 (2020).

47. Hedenus, F., Karlsson, S., Azar, C. & Sprei, F. Cost-effective energy carriers for transport – The

role of the energy supply system in a carbon-constrained world. *International Journal of Hydrogen Energy* **35**, 4638–4651 (2010).

48. Bertram, C. *et al.* Energy system developments and investments in the decisive decade for the Paris Agreement goals. *Environ. Res. Lett.* **16**, 074020 (2021).
49. Labandeira, X., Labeaga, J. M. & López-Otero, X. A meta-analysis on the price elasticity of energy demand. *Energy Policy* **102**, 549–568 (2017).
50. Dimitropoulos, A., Oueslati, W. & Sintek, C. The rebound effect in road transport: A meta-analysis of empirical studies. *Energy Economics* **75**, 163–179 (2018).
51. Persson, T. A., Azar, C., Johansson, D. & Lindgren, K. Major oil exporters may profit rather than lose, in a carbon-constrained world. *Energy Policy* **35**, 6346–6353 (2007).
52. Tanaka, K., Lund, M. T., Aamaas, B. & Berntsen, T. Climate effects of non-compliant Volkswagen diesel cars. *Environ. Res. Lett.* **13**, 044020 (2018).
53. Tanaka, K., Johansson, D. J. A., O'Neill, B. C. & Fuglestvedt, J. S. Emission metrics under the 2 °C climate stabilization target. *Climatic Change* **117**, 933–941 (2013).
54. Tanaka, K. *et al.* Cost-effective implementation of the Paris Agreement using flexible greenhouse gas metrics. (2020).
55. Melnikova, I., Ciais, P., Boucher, O. & Tanaka, K. Assessing carbon cycle projections from complex and simple models under SSP scenarios. *Climatic Change* **176**, 168 (2023).
56. Kvale, K. *et al.* Carbon Dioxide Emission Pathways Avoiding Dangerous Ocean Impacts. *Weather, Climate, and Society* **4**, 212–229 (2012).
57. Nicholls, Z. R. J. *et al.* Reduced Complexity Model Intercomparison Project Phase 1: introduction and evaluation of global-mean temperature response. *Geoscientific Model Development* **13**, 5175–5190 (2020).
58. Goll, D., Joetzjer, E., Huang, M. & Ciais, P. Low Phosphorus Availability Decreases Susceptibility of Tropical Primary Productivity to Droughts. *Geophysical Research Letters* **45**, 8231–8240 (2018).
59. Sun, Y. *et al.* Global evaluation of the nutrient-enabled version of the land surface model ORCHIDEE-CNP v1.2 (r5986). *Geoscientific Model Development* **14**, 1987–2010 (2021).

60. Friedlingstein, P. *et al.* Global Carbon Budget 2019. *Earth System Science Data* **11**, 1783–1838 (2019).

61. Azar, C., Lindgren, K., Larson, E. & Möllersten, K. Carbon Capture and Storage From Fossil Fuels and Biomass – Costs and Potential Role in Stabilizing the Atmosphere. *Climatic Change* **74**, 47–79 (2006).

62. Azar, C., Johansson, D. J. A. & Mattsson, N. Meeting global temperature targets—the role of bioenergy with carbon capture and storage. *Environ. Res. Lett.* **8**, 034004 (2013).

63. Wilson, C. *et al.* Evaluating process-based integrated assessment models of climate change mitigation. *Climatic Change* **166**, 3 (2021).

64. Welsby, D., Price, J., Pye, S. & Ekins, P. Unextractable fossil fuels in a 1.5 °C world. *Nature* **597**, 230–234 (2021).

65. Daioglou, V., Doelman, J. C., Wicke, B., Faaij, A. & van Vuuren, D. P. Integrated assessment of biomass supply and demand in climate change mitigation scenarios. *Global Environmental Change* **54**, 88–101 (2019).

66. Daioglou, V. *et al.* Greenhouse gas emission curves for advanced biofuel supply chains. *Nature Clim Change* **7**, 920–924 (2017).

67. Hanssen, S. V. *et al.* The climate change mitigation potential of bioenergy with carbon capture and storage. *Nat. Clim. Chang.* **10**, 1023–1029 (2020).

68. Wang, J. *et al.* Temperature Changes Induced by Biogeochemical and Biophysical Effects of Bioenergy Crop Cultivation. *Environmental Science & Technology* (2023) doi:10.1021/acs.est.2c05253.

69. Merfort, L. *et al.* Bioenergy-induced land-use-change emissions with sectorally fragmented policies. *Nat. Clim. Chang.* (2023) doi:10.1038/s41558-023-01697-2.

70. Harmsen, M. J. H. M. *et al.* Data for long-term marginal abatement cost curves of non-CO2 greenhouse gases. *Data in Brief* **25**, 104334 (2019).

71. IRENA. *Renewable Power Generation Costs in 2020, International Renewable Energy Agency, Abu Dhabi.* (2021).

72. NREL. *'2021 Annual Technology Baseline.' Golden, CO: National Renewable Energy*

*Laboratory.* (2021).

73. Cost and Performance Characteristics of New Generating Technologies, Annual Energy Outlook 2022. 4.

74. Luderer, G. *et al.* Impact of declining renewable energy costs on electrification in low-emission scenarios. *Nat Energy* **7**, 32–42 (2022).

75. Riahi, K. *et al.* Cost and attainability of meeting stringent climate targets without overshoot. *Nat. Clim. Chang.* **11**, 1063–1069 (2021).

76. Lehtveer, M., Mattsson, N. & Hedenus, F. Using resource based slicing to capture the intermittency of variable renewables in energy system models. *Energy Strategy Reviews* **18**, 73–84 (2017).

77. Sherwood, S. C. *et al.* An Assessment of Earth's Climate Sensitivity Using Multiple Lines of Evidence. *Rev. Geophys.* **58**, (2020).
